# Kif15 orchestrates neuronal–microglial communication *via* CX3CL1 to impede nerve regeneration

**DOI:** 10.1016/j.jbc.2026.113090

**Published:** 2026-04-28

**Authors:** Ronghua Wu, Wei Zhang, Xiaowei Qian, Zhangji Dong, Xiaomei He, Siming Zhang, Taoran Chen, Liu Yang, Yan Liu, Mei Liu

**Affiliations:** 1 Jiangsu Key Laboratory of Tissue Engineering and Neuroregeneration, Key Laboratory of Neuroregeneration of Ministry of Education, Co-innovation Center of Neuroregeneration, Nantong University, Nantong, China; 2 Department of Neurosurgery, Affiliated Hospital of Nantong University, Nantong, China

**Keywords:** kif15, sciatic nerve injury, snRNA-seq, CX3CL1-CX3CR1, neuronal-microglial communication

## Abstract

Kinesins, a class of microtubule (MT)-dependent molecular motors, regulate MT dynamics and MT-mediated transport. We previously identified Kif15 (kinesin-12) as a key player in axonal growth by modulating MT remodeling during neuronal development, and more recently, its involvement in protein localization. In this study, we observed that Kif15 knockout (Kif15 KO) mice exhibited accelerated functional recovery after sciatic nerve injury. To investigate the cellular responses underlying this enhanced recovery after axotomy, spinal cord tissues from the injured regions were collected for single-nucleus RNA sequencing (snRNA-seq). The snRNA-seq results revealed differential genes expression in neurons, indicating a neuroprotective shift in Kif15 KO mice, and in microglia, where a repair-promoting and synapse-modulating profile was observed. Notably, the CX3CL1-CX3CR1 signaling pathway, critical for neuronal-microglial communication, was downregulated in Kif15 KO mice compared to wild-type controls. Further molecular analysis indicated that Kif15 facilitated the expression and localization of neuronal CX3CL1, which, in turn, influenced microglial function *via* the receptor CX3CR1. Our findings highlight a novel role for Kif15 in regulating neuronal-microglial communication through modulation of CX3CL1 signaling.

Peripheral nerve injuries, such as those affecting the sciatic nerve, induce Wallerian degeneration and activate regenerative processes, though functional recovery is often incomplete (1). Beyond local damage, these injuries initiate a cascade of events within the central nervous system, activating spinal microglia and astrocytes, which together modulate a microenvironment that influences both neuronal survival and axonal regeneration (2, 3). At the cellular level, axonal injury generates damage signals that retrogradely travel to the neuronal cell body, where they modify transcriptional programs, ultimately determining whether the neuron will regenerate or undergo degeneration (4, 5). While axons in the peripheral nervous system possess intrinsic regenerative potential (6), the precise molecular mechanisms governing long-distance communication between the injury site and the neuronal soma remain unclear.

This long-distance signaling relies on axonal transport. Given the distance between the lesion and the neuronal soma, motor proteins that transport cargo along microtubules are crucial in coordinating the injury response (7). Kif15 (kinesin-12), a plus-end-directed microtubule motor traditionally associated with cell division (8), has emerged as a key candidate regulator of neuronal injury responses. Although evidence suggests kinesin-12 is involved in neuronal plasma membrane trafficking (9), most studies in post-mitotic neurons have focused on its role in regulating microtubule dynamics and organization (10, 11). We previously showed that Kif15 depletion accelerates growth rates in primary neurons (11). However, the precise physiological roles of Kif15 *in vivo*, particularly in the context of peripheral nerve injury, remain to be fully understood.

We utilized the CRISPR/Cas9 system to generate *Kif15*^*−/−*^ mice. Throughout the breeding and maintenance of these mice, no significant differences in overall development or reproductive performance were observed compared to wild-type controls. While no major histological changes were found between *Kif15*^*−/−*^ and *Kif15*^*+/+*^ mice in tissues such as lung, kidney, liver, heart, and brain, a slight delay in testis and spleen maturation was noted in *Kif15*^*−/−*^ mice, suggesting that Kif15 is related to cell division and its absence may impair cell proliferation (12). Further investigations into neuronal function revealed that *Kif15*^*−/−*^ female mice exhibited depressive behaviors in behavioral tests (13), and we further identified that Kif15 modulates emotional regulation through control of PSD95 transport (14). These observations suggest that Kif15 may influence neural activity or regulation *via* its role in microtubule-based transport.

Bulk RNA sequencing averages transcriptomic signals across heterogeneous cell populations (*e.g.*, microglia, neurons, astrocytes, and infiltrating immune cells) in spinal cord injury (SCI) tissues, limiting its ability to reveal cell-type-specific transcriptional changes. The advent of single-cell RNA sequencing (scRNA-seq) has revolutionized tissue analysis by enabling high-resolution deconstruction of heterogeneous tissues at the cellular level (15). As shown in previous studies (16), scRNA-seq can result in substantial neuronal loss. In contrast, single-nucleus RNA sequencing (snRNA-seq) offers distinct advantages for nervous system studies, as it preserves over 85% of neuronal subpopulations (17). The complex morphology of neurons, with their extensive and fragile axons and dendrites, makes them highly susceptible to mRNA loss and transcriptional biases during the enzymatic digestion required for scRNA-seq (18). snRNA-seq overcomes this limitation, providing a more accurate transcriptional profile of neuronal cell bodies. Consequently, we employed snRNA-seq to explore the molecular mechanisms by which Kif15 influences nerve repair at both the cellular and molecular levels.

Here, we demonstrate that genetic ablation of Kif15 significantly enhances functional recovery following sciatic nerve crush. Through integrative snRNA-seq analysis, we show that Kif15 deficiency alters the course of injury-induced microglial activation and shifts the molecular profile of motor neurons towards a pro-regenerative state. These findings establish Kif15 as a novel negative regulator of peripheral nerve regeneration and highlight its potential as a therapeutic target for enhancing functional recovery after nerve injury.

## Results

### Kif15 Deficiency Accelerates Functional Recovery and Axonal Regeneration After Sciatic Nerve Injury

**Figure 1:**
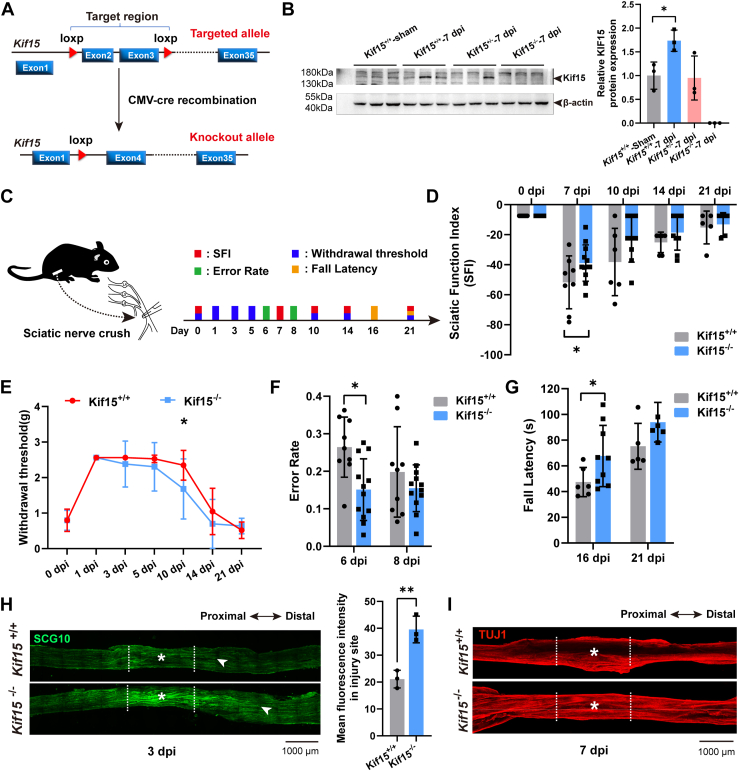
**Kif15 Deficiency Accelerates Functional Recovery and Axonal Regeneration After Sciatic Nerve Injury.***A*, the diagram illustrates the gene-targeting strategy used to produce the Kif15 knockout mice in C57BL/6J background. *B*, Western blot analysis of Kif15 expression in the spinal cord of *Kif15*^*+/+*^, *Kif15*^*+/−*^ and *Kif15*^*−/−*^ mice after sham or nerve injury (7 dpi). Relative Kif15 protein expression normalized to β-actin levels. Data are presented as mean ± SE. Statistical significance was determined using a two-tailed unpaired Student's *t* test, ∗*p* < 0.05. *C*, Schematic illustration of sciatic nerve injury (SNI) and function test of the timeline. Ten-week-old *Kif15*^*+/+*^ or *Kif15*^*−/−*^ mice were crush injured in the *right* sciatic nerve and sham-operated in the *left* sciatic nerve. *D*, Sciatic Functional Indexes (SFI) recorded at 0, 7, 10, 14, 21 dpi, n = 6 to 12 mice per group. The data were shown as mean ± SD. Data were analyzed using two-way ANOVA followed by Bonferroni's *post hoc* test, ∗*p* < 0.05. *E*, the changes in mechanical withdrawal threshold at 0, 1, 3, 5, 10, 14, 21 dpi. Animals developed significant mechanical allodynia at 10 dpi, n = 10 to 12 mice per group. The data were shown as mean ± SD. Data were analyzed using two-way ANOVA followed by Bonferroni's *post hoc* test, ∗*p* < 0.05. *F*, Error rate analyses of hindlimb movements at 6 dpi and 8 dpi (*Kif15*^*+/+*^ group, n = 12 mice; *Kif15*^*−/−*^ group, n = 9 mice). The data was shown as mean ± SD. Data were analyzed using unpaired Student’s *t* test, ∗*p* < 0.05. *G*, Latency to fall and fall times in the rotarod test at 16 dpi (*Kif15*^*+/+*^ group, n = 9 mice; *Kif15*^*−/−*^ group, n = 6 mice) and 21 dpi (*Kif15*^*+/+*^ group, n = 5 mice; *Kif15*^*−/−*^ group, n = 5 mice). The data were shown as mean ± SD. Data were analyzed using unpaired Student’s *t* test, ∗*p* < 0.05. *H*, *Left panel*, representative immunostaining images of SCG10 (a marker for regenerating axons) in *Kif15*^*+/+*^ and *Kif15*^*−/−*^ mice sciatic nerve at 3 dpi, the *asterisk* indicates the injured area, scale bar = 1000 μm. *Right panel*, statistical analysis. The data was shown as mean ± SD, *Kif15*^*−/−*^ group vs the *Kif15*^*+/+*^ group, two-tailed unpaired Student’s *t* test, n = 3 mice, ∗∗*p* < 0.01. *I*, Tissue clearing and Tuj1-immunostaining result of the sciatic nerves from *Kif15*^*+/+*^ and *Kif15*^*−/−*^ mice at 7 dpi. The *asterisk* indicates the injured area, scale bar = 1000 μm.

To investigate the role of Kif15 in nerve regeneration *in vivo*, *Kif15*^*−/−*^ C57BL/6J mice were generated using CRISPR/Cas9-mediated gene targeting (Fig. 1*A*). A sciatic nerve crush injury was then performed on adult mice. Initial analysis of Kif15 protein expression in the L4-L6 spinal cord at 7 days post-injury (dpi) revealed a significant increase in *Kif15*^*+/+*^ mice, no notable change in *Kif15*^*+/−*^ mice, and complete absence in *Kif15*^*−/−*^ mice (Fig. 1*B*), indicating that sciatic nerve injury (SNI) induced an upregulation of Kif15 protein in the spinal cord segment containing the neuronal cell bodies. Following sciatic nerve crush, functional recovery was monitored according to the schedule in Figure 1*C*. Compared to *Kif15*^*+/+*^ mice, *Kif15*^*−/−*^ mice showed a significant improvement in the sciatic nerve function index (SFI) at 7 dpi (Fig. 1*D*), indicating enhanced motor function recovery. Mechanical allodynia, assessed using the withdrawal threshold test, was significantly reduced in *Kif15*^*−/−*^ mice at 10 dpi (Fig. 1*E*). The error rate assay for hindlimb movements showed a notable decrease in *Kif15*^*−/−*^ mice at 6 dpi (Fig. 1*F*). Additionally, motor coordination and balance, assessed using the rotarod test, revealed a significant increase in latency time for *Kif15*^*−/−*^ mice at 16 dpi (Fig. 1*G*). Histological analysis of the injured sciatic nerves at 3 dpi and 7 dpi demonstrated improved regeneration. Immunostaining with SCG10 antibody at 3 dpi (Fig. 1*H*) showed more SCG10-positive regenerating axons in *Kif15*^*−/−*^ mice compared to *Kif15*^*+/+*^ mice. Tissue clearing and TUJ1 immunostaining further confirmed a marked increase in TUJ1-positive axons in injured nerves of *Kif15*^*−/−*^ mice (Fig. 1*I*). These results indicate significant improvements in both the neurological morphology and function of injured axons in *Kif15*^*−/−*^ mice.

### Kif15 knockout induces a transient and resolving microglial activation in the spinal cord

**Figure 2:**
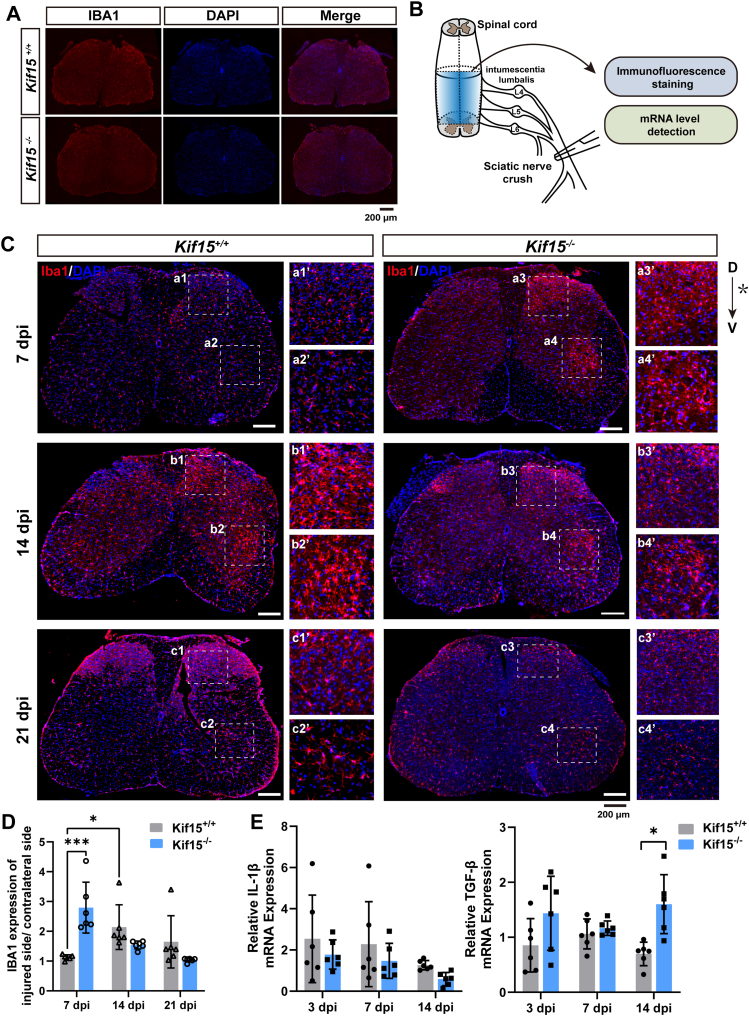
**Activation of microglia in Kif15^−/−^ mice was earlier than that of in Kif15^+/+^ mice.***A*, immunohistochemistry staining of IBA1(*red*) and DAPI (*blue*) in spinal cord lumbar segments of *Kif15*^*+/+*^and *Kif15*^*−/−*^ mice. Scale bar = 200 μm. *B*, schematic diagram of spinal cord lumbar segments after SNI. *C*, immunohistochemistry staining of IBA1(*red*) and DAPI (*blue*) in spinal cord lumbar segments at 7, 14, 21 dpi. Panels a1′– a4′, b1′– b4′, c1′– c4′ are magnifications of the *white squares* in panels a a1–a4, b b1–b4, and c c1–c4, respectively. The *asterisk* represents the nerve injured side, *D* indicates dorsal, *V* indicates ventral, scale bar = 200 μm. *D*, expression of IBA1 in spinal cord lumbar segments at 7, 14, 21 dpi. The ratio of average fluorescence intensity at the nerve injured side and the contralateral side, were used to plot the bar graph. The data were shown as mean ± SD, *Kif15*^*−/−*^ group vs the *Kif15*^*+/+*^ group, two-tailed unpaired Student’s *t* test, n = 6 mice, ∗*p* < 0.05, ∗∗∗*p* < 0.001. *E*, *Left* panel, qRT-PCR results of *IL-1β* expression in spinal cord lumbar segments at 3, 7, 14 dpi. *Right panel*, qRT-PCR results of *TGF-β* expression in spinal cord lumbar segments at 3, 7, 14 dpi. The data was shown as mean ± SD. Data were analyzed using two-way ANOVA followed by Tukey’s *post hoc* test (*p* < 0.05), n = 6 mice, ∗*p* < 0.05.

Following SNI, damage signals are retrogradely transported to neuronal cell bodies in the spinal cord, triggering cellular responses (19). The nature of these responses, which vary across different cell types, ultimately determines whether neural degeneration or regeneration occurs. To investigate whether Kif15 knockout alters cellular responses in the spinal cord after SNI, we performed immunostaining to assess changes in neurons, astrocytes, and microglia. First, spinal cord tissues from 10-week-old sham-injured mice were analyzed. Immunostaining results revealed no significant differences in the expression patterns of IBA1, NeuN, and GFAP between *Kif15*^*+/+*^ and *Kif15*^*−/−*^ mice (Figs. 2*A* and S1*A*).

Subsequently, spinal cords from sciatic nerve-injured mice were examined. The results showed that IBA1-positive microglia were significantly activated in the dorsal corticospinal tract, motor neurons of the ventral horn, and lateral motor neuron pools innervating flexor and extensor muscles on the ipsilateral side compared to the contralateral side. Notably, compared to *Kif15*^*+/+*^ mice, IBA1-positive microglia in *Kif15*^*−/−*^ mice were activated earlier and resolved more quickly (Fig. 2, *B*–*D*). As shown in the right panel of Figure 2*C*, microglia surrounding the cell bodies of the sciatic nerve in *Kif15*^*−/−*^ mice were strongly activated at 7 dpi, followed by a gradual decrease at 14 dpi and 21 dpi. In contrast, microglia in *Kif15*^*+/+*^ mice were weakly activated at 7 dpi, strongly activated at 14 dpi, and then decreased at 21 dpi (Fig. 2*C*, left panel). No significant alterations were observed in the expression of neuronal (NeuN) or astrocytic (GFAP) markers (Fig. S1, *B* and *C*).

To further characterize the functional phenotype of the activated microglia and determine whether Kif15 knockout promotes a pro-inflammatory or anti-inflammatory environment, we measured the mRNA expression levels of the pro-inflammatory factor IL-1β and the anti-inflammatory factor TGF-β in the injured spinal cord at 3, 7, and 14 dpi. The results showed that *Kif15* knockout reduced the expression of *IL-1β* and increased the expression of *TGF-β* (Fig. 2*E*). These results suggest that Kif15 deficiency leads to transient microglial activation with a tendency towards M2 polarization during the early pathological stage, potentially creating a more favorable environment for tissue repair.

### Single-nucleus RNA-seq reveals attenuated neuron-to-microglia signaling in Kif15 KO mice

**Figure 3:**
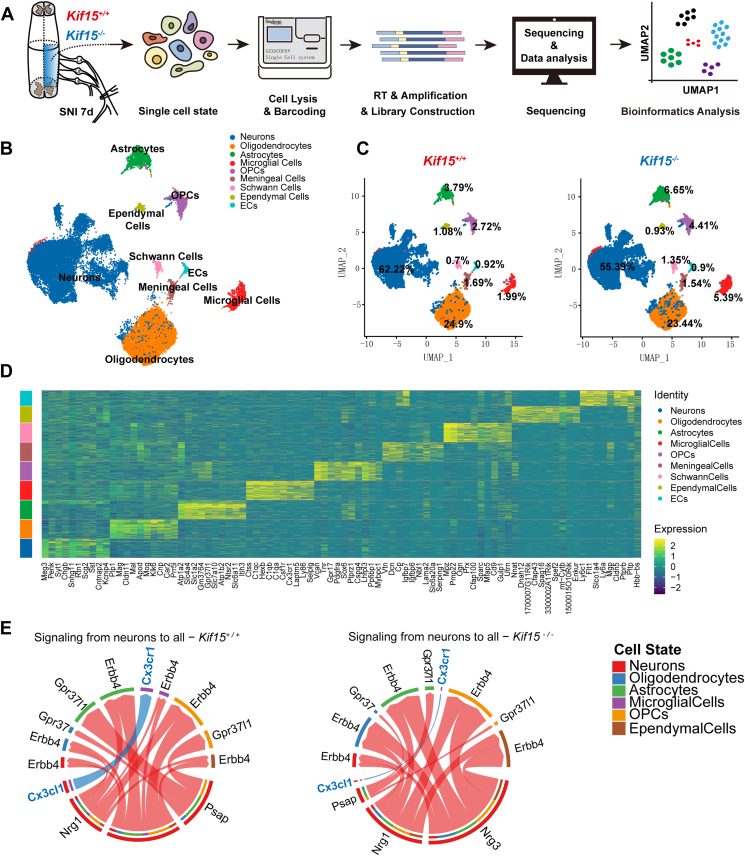
**Single-nucleus RNA-Seq reveals attenuated neuron-to-microglia signaling in Kif15 KO mice.***A*, workflow for single-cell collection, sequencing, and analysis. *B*, UMAP visualization plot of 26603 spinal cord cells sequenced from all samples, color-coding defined 9 major cell types based on signature gene expression. *C*, the proportion of each cell type in *Kif15*^*+/+*^ and *Kif15*^*−/−*^ mice at 7 dpi. *D*, Heatmap of the average expression of top 10 differentially expressed genes among nine subclusters of spinal cord cells from *Kif15*^*+/+*^ mice and *Kif15*^*−/−*^ mice. *Yellow* color represents high gene expression, and the *navy blue* represents low expression. *E*, Cellchat analysis revealed that the communication of the CX3CL1-CX3CR1 signal pathway from neurons to microglia was markedly attenuated.

We next explored the molecular mechanisms underlying the enhanced functional recovery observed in *Kif15*^*−/−*^ mice following sciatic nerve crush injury. Given the cellular diversity in the spinal cord, which comprises multiple cell types with distinct transcriptional profiles, we employed snRNA-seq to dissect cellular heterogeneity and identify cell-type-specific transcriptomic changes associated with Kif15 deficiency. Spinal cord tissues from the L4-L6 region (5 mm in length) of *Kif15*^*−/−*^ and *Kif15*^*+/+*^ mice at 7 dpi were subjected to snRNA-seq analysis (Fig. 3*A*). A total of 26,603 cells were sequenced, and nine distinct cell types were defined based on the expression of cell-specific markers, as previously described (20) (Fig. 3*B*), with detailed gene markers listed in the methods. Differential expression analysis revealed mutually exclusive sets of genes characteristic of specific cell lineages, frequently including well-established markers for particular cell types. The top 10 most differentially expressed markers for each cluster are provided in Figure 3*D*.

The proportions of each cell type were quantified, revealing a significant increase in microglial cells (from 1.99% in *Kif15*^*+/+*^ mice to 5.39% in *Kif15*^*−/−*^ mice), which was consistent with the Iba1 immunostaining results shown in Figure 2*C*. In contrast, the proportions of other cell types did not differ significantly between *Kif15*^*−/−*^ and *Kif15*^*+/+*^ mice (Fig. 3*C*).

Unlike the central nervous system, the peripheral nervous system has the capacity to regenerate. After a sciatic nerve crush, injury signals are retrogradely transmitted to the motor neuron cell bodies located in the anterior horn of the spinal cord or to the sensory neuron cell bodies in the dorsal root ganglion. In response, the neuronal cell bodies alter their gene expression profiles and enter a "repair state" (21). To investigate cell-cell communication, we utilized the CellChat 1.5.0 package (22). Given that injured neurons release signaling molecules that activate surrounding microglia, we focused on signaling pathways from neurons to microglia. The results showed that the CX3CL1-CX3CR1 axis, which regulates neuron–microglia communication, was significantly attenuated in *Kif15*^*−/−*^ neurons compared to *Kif15*^*+/+*^ neurons (Fig. 3*E*).

### Loss of Kif15 enhances the survival and repairable capacities of spinal neurons to injury

**Figure 4:**
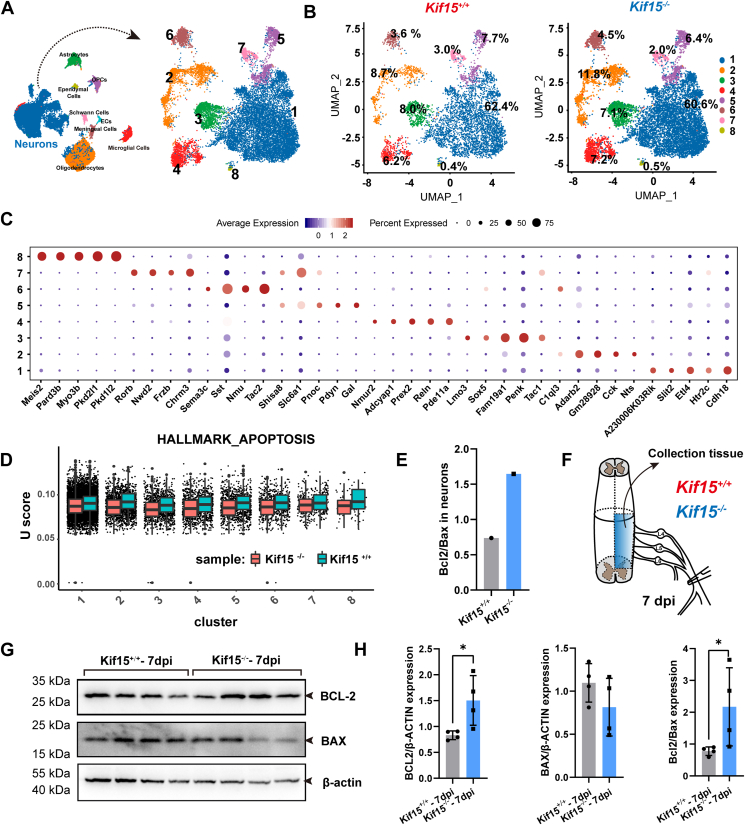
**Loss of Kif15 enhances the intrinsic anti-apoptosis of spinal neurons to injury.***A*, UMAP plot showing eight neuronal clusters (subtypes). *B*, The proportion of each neuronal subtype in *Kif15*^*+/+*^ and *Kif15*^*−/−*^ mice at 7 dpi. *C*, Dotplot illustrating the normalized mean expression of top 5 signature genes for each neuronal cluster. *D*, Comparison of UCell score in neuronal clusters apoptosis between *Kif15*^+/+^ and *Kif15*^−/−^ mice after sciatic nerve injury. *E*, relative ratio of Bcl2/Bax in neurons between *Kif15*^+/+^ and *Kif15*^−/−^ mice after sciatic nerve injury. *F*, schematic diagram of organization collection. *G*, Western blotting assays indicated the expression level of BCL2 and BAX in the spinal cord of *Kif15*^*+/+*^ and *Kif15*^*−/−*^ mice at 7 dpi. *H*, statistical analysis of *panel G*. The data were shown as mean ± SD, *Kif15*^*−/−*^ group vs the *Kif15*^*+/+*^ group, two-tailed unpaired Student’s *t* test, n = 4 mice, ∗*p* < 0.05.

The neuronal population was the most abundant cell type detected in the spinal cord following sciatic nerve crush, as determined by snRNA-seq analysis, with a total of eight distinct neuronal subpopulations identified based on differential marker genes. The results indicated that the proportions of each subgroup remained similar, suggesting that the injury did not induce significant shifts in cell population distribution (Fig. 4, *A*–*C*). Given that Figure 1, *H* and *I* demonstrated a greater presence of regenerative nerve fibers in the crush area of *Kif15*^*−/−*^ mice, we examined whether neuronal apoptosis was reduced in these mice after injury. The results showed a significant decrease in pan-apoptotic signals across all eight spinal neuronal subtypes in *Kif15*^*−/−*^ mice, with the Bcl2/Bax ratio increasing by 100% at the transcript level (Fig. 4, *D* and *E*). Further validation through Western blot analysis confirmed a significant increase in BCL2 protein expression and a slight decrease in BAX expression, resulting in a marked elevation in the BCL2/BAX ratio in *Kif15*^*−/−*^ mice, consistent with the snRNA-seq findings (Fig. 4, *F*–*H*).

### Microglia in Kif15 KO mice obtain a pro-repair, synapse-modulating phenotype

**Figure 5:**
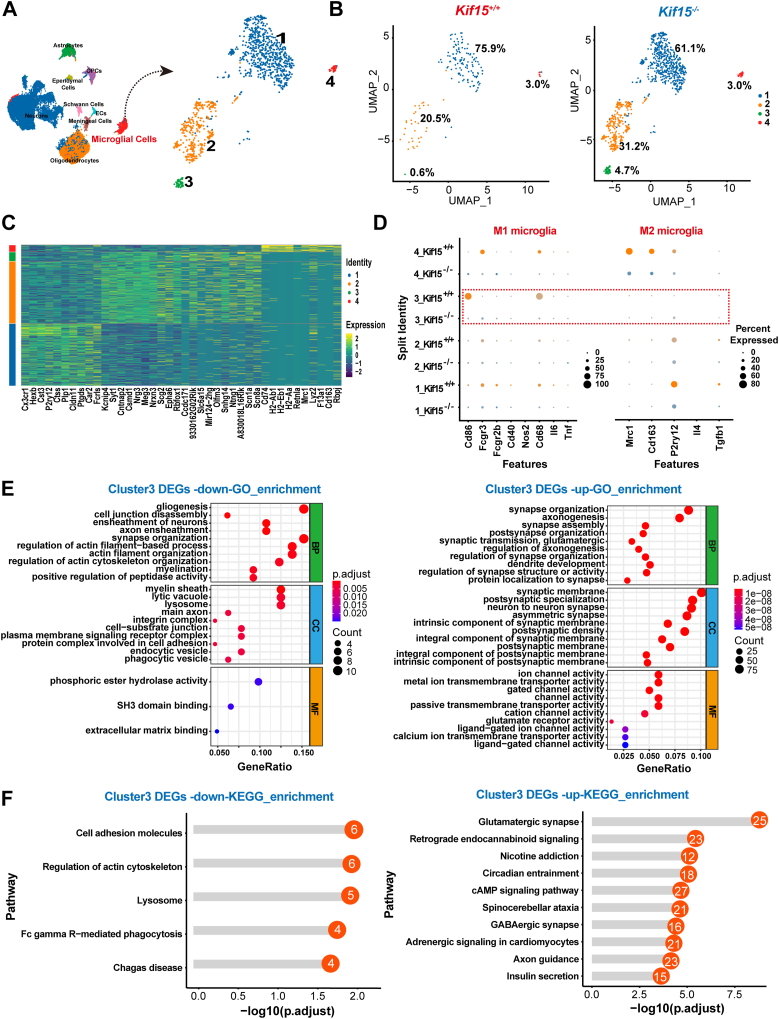
**Microglia in Kif15 KO mice adopt a pro-repair, synapse-modulating phenotype.***A*, UMAP plot showing four microglia clusters (subtypes). *B*, The proportion of each microglia subtype in *Kif15*^*+/+*^ and *Kif15*^*−/−*^ mice at 7 dpi. *C*, heatmap of the average expression of the top 10 differentially expressed genes among four microglia subtypes. *Yellow* color represents high gene expression, and the *navy blue* represents low expression. *D*, dotplot illustrating the normalized mean expression of M1/M2 microglia marker genes among four subtypes between *Kif15*^*+/+*^ mice and *Kif15*^*−/−*^ mice. *E*, *left* panel, Gene ontology (GO) analysis of the downregulated DEGs in cluster 3; *Rightpanel*, GO analysis of the upregulated DEGs in cluster 3. *F*, *Leftpanel*, Kyoto Encyclopedia of Genes and Genomes (KEGG) enrichment analysis of the downregulated DEGs in cluster 3; *Rightpanel*, KEGG analysis of the upregulated DEGs in cluster 3.

We analyzed the changes in microglial proportion and found a significant increase in the microglial ratio in the spinal cord of *Kif15*^*−/−*^ mice (5.39%), compared to *Kif15*^*+/+*^ mice (1.99%) (Fig. 3*C*). This finding aligns with the Iba1 immunostaining results shown in Figure 2, which indicated enhanced microglial activation in *Kif15*^*−/−*^ mice at 7 dpi. The microglia were classified into four subgroups (Fig. 5*A*), with the distribution of each subgroup presented in Figure 5*B*. It is well established that microglial activation in response to injury or pathological conditions leads to polarization into pro-inflammatory (M1) or anti-inflammatory (M2) states (23). To explore whether microglial polarization was altered in the spinal cords of *Kif15*^*−/−*^ mice, we compared the expression levels of M1 and M2 markers across the four subgroups. The results showed a significant decrease in M1-type markers, while M2-type markers were also reduced, except for P2RY12 expression, which increased in subgroup 3 (Fig. 5, *C* and *D*).

Furthermore, the proportion of subgroup 3 microglia was nearly seven times greater in *Kif15*^*−/−*^ mice (4.7%) compared to wild-type mice (0.6%) (Fig. 5*B*). We then selected the upregulated and downregulated differentially expressed genes (DEGs) in cluster 3 microglia for Gene Ontology (GO) and Kyoto Encyclopedia of Genes and Genomes (KEGG) enrichment analyses. GO enrichment analysis of downregulated genes revealed significant enrichment in cellular components (CC) related to the myelin sheath, molecular functions (MF) associated with phosphoric ester hydrolase activity, and biological processes (BP) linked to gliogenesis. Conversely, upregulated genes in this cluster were enriched in CC related to synaptic membranes, MF associated with ion channel activity, and BP related to synapse organization (Fig. 5*E*). KEGG enrichment analysis showed a decrease in pathways associated with the cytoskeleton and lysosomes, and an increase in pathways related to glutamatergic synapses (Fig. 5*F*). These results suggest that activated microglia in *Kif15*^*−/−*^ mice are more involved in synaptic remodeling rather than the clearance and enzymatic degradation processes.

### Kif15 governs the expression and subcellular localization of the neuronal chemokine CX3CL1

**Figure 6:**
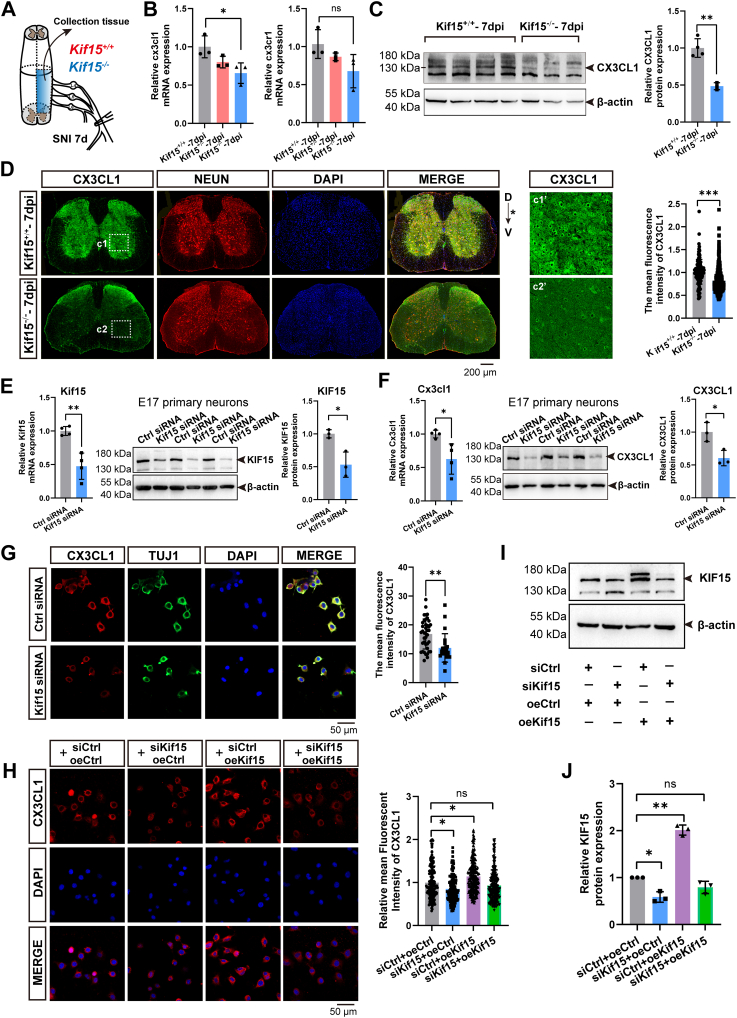
**Kif15 governs the expression and subcellular localization of the neuronal chemokine.** CX3CL1 *A*, schematic diagram of organization collection. *B*, qRT-PCR results of Cx3cl1 and Cx3cr1 in Kif15^+/+^, Kif15^+/−^ and Kif15^−/−^ mice at 7 dpi. The data was shown as mean ± SD. Data were analyzed using unpaired Student’s *t* test, ∗*p* < 0.05. *C*, *left panel*, western blotting assays indicated the expression level of CX3CL1 in spinal cord of Kif15^+/+^ and Kif15^−/−^ mice at 7 dpi. Right panel, statistical analysis. The data was shown as mean ± SD, two-tailed unpaired Student’s *t* test, (*Kif15*^*+/+*^ group, n = 4 mice, *Kif15*^*−/−*^ group, n = 3 mice), ∗*p* < 0.05. *D*, *left panel*, immunohistochemistry staining of CX3CL1 in spinal cord from Kif15^+/+^ and Kif15^−/−^ mice at 7 dpi, scale bar = 200 μm. Panels c1′– c2′ are magnifications of the *white squares* in panels c1–c2, respectively. *Rightpanel*, statistical analysis. The asterisk represents the nerve injured side, D indicates dorsal, V indicates ventral, the data was shown as mean ± SD. Data were analyzed using unpaired Student’s *t* test, ∗∗∗*p* < 0.001. *E*, qRT-PCR and western blotting results of Kif15 in E17 neurons after Ctrl or Kif15 siRNA treatment. The data was shown as mean ± SD. Data were analyzed using two-tailed unpaired Student’s *t* test, ∗*p* < 0.05, ∗∗*p* < 0.01. *F*, qRT-PCR and western blotting results of Cx3cl1 in E17 neurons after Ctrl or Kif15 siRNA treatment. The data were shown as mean ± SD. Data were analyzed using unpaired Student’s *t* test, ∗*p* < 0.05. *G*, *leftpanel*, immunohistochemistry staining of CX3CL1 in N2a cells after Ctrl or Kif15 siRNA treatment. *Right panel*, statistical analysis. The data were shown as mean ± SD. Data were analyzed using two-tailed unpaired Student’s *t* test, ∗∗*p* < 0.01. *H*, *Leftpanel*, immunofluorescence staining of CX3CL1 in N2a cells after transfection with Ctrl siRNA, Kif15 siRNA, oeCtrl, or oeKif15 treatments. Scale bar = 50 μm. *Right panel*, statistical analysis. The data were shown as mean ± SD. Data were analyzed using one-way ANOVA followed by Tukey’s *post hoc* test, ∗*p* < 0.05. *I*, Western blotting assays showing CX3CL1 and KIF15 expression levels in N2a cells after Ctrl siRNA, Kif15 siRNA, control overexpression (oeCtrl), or Kif15 overexpression (oeKif15) treatments. *J*, Statistical analysis of *panel I*. The data was shown as mean ± SD. Data were analyzed using one-way ANOVA followed by Tukey’s *post hoc* test, ∗*p* < 0.05, ∗∗*p* < 0.01.

Based on the data presented in Figure 3*E*, we hypothesize that the CX3CL1 molecule is reduced in Kif15-depleted neurons, which leads to the activation of the CX3CR1 pathway in microglia. To test this hypothesis, sciatic nerve crush models were established in *Kif15*^*+/+*^, *Kif15*^*+/−*^, and *Kif15*^*−/−*^ mice. After 7 days, spinal cord segments from the injured side were collected to assess the mRNA and protein levels of CX3CL1 and CX3CR1 (Fig. 6*A*). The results showed that Kif15 depletion significantly downregulated both the mRNA and protein expression of CX3CL1, with no significant change observed in the mRNA expression of CX3CR1 (Fig. 6, *B* and *C*). Immunostaining further confirmed a notable reduction in CX3CL1 expression in neurons (Fig. 6*D*).

Next, we examined the correlation between Kif15 and CX3CL1 expression using *Kif15* siRNA in cultured neurons and Neuro2a cells. Kif15 knockdown resulted in a decrease in CX3CL1 expression (Fig. 6, *E* and *F*), and immunostaining revealed a significant reduction in the membrane localization of CX3CL1 (Fig. 6*G*).

To investigate whether Kif15 affects the membrane localization of CX3CL1, we performed a rescue experiment in Neuro-2a cells by overexpressing Kif15. The results, shown in Figure 6, *H*–*J*, demonstrated that compared to the control group, the fluorescent intensity of CX3CL1 on the cell membrane decreased by 25% in Kif15 siRNA-treated cells, while it increased by 35% in Kif15 plasmid-overexpressing cells. In cells treated with both siKif15 and Kif15 plasmid, no significant changes in intensity were observed. These results suggest that Kif15 modulates both the expression and membrane localization of CX3CL1.

In the central nervous system, CX3CL1 is constitutively expressed by neurons and plays a critical role in the communication between neurons and innate immune cells, particularly microglia (24, 25, 26). Although CX3CL1 is predominantly membrane-bound, it can be cleaved by ADAM10 or ADAM17 to generate a soluble, diffusible form (27, 28). As a transmembrane protein, membrane-bound CX3CL1 exists on the neuronal surface and serves as an adhesion molecule, directly binding to microglia expressing CX3CR1 to mediate physical adhesion. The CX3CL1-CX3CR1 axis is essential for maintaining microglial homeostasis and synaptic monitoring under steady-state conditions (25). In contrast, the soluble form, sCX3CL1, detaches from the membrane and functions as a chemokine, exerting chemotactic effects to recruit microglia to sites of injury or inflammation (28).

**Figure 7:**
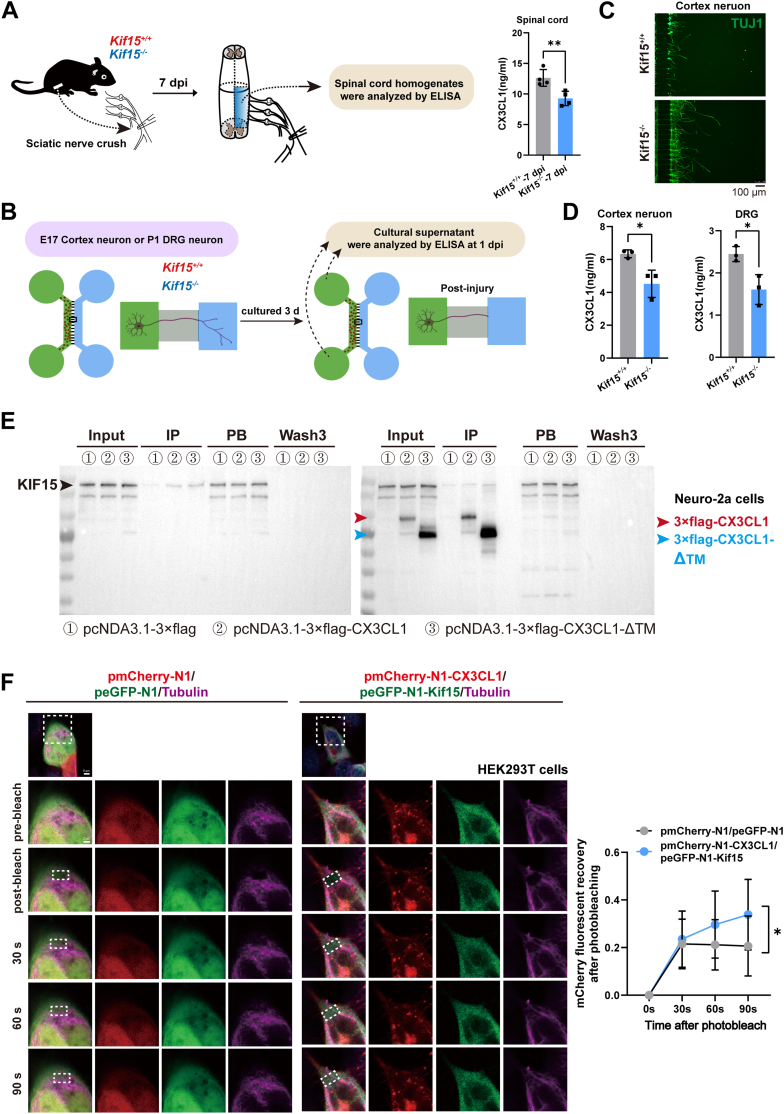
**KIF15 mediates the transport of CX3CL1 on microtubules.***A*, *left panel*, schematic diagram of the experimental timeline. Sciatic nerve crush was performed in *Kif15*^*+/+*^ and *Kif15*^*−/−*^ mice, and spinal cord homogenates were collected at 7 dpi for ELISA analysis. *Right panel*, ELISA quantification of CX3CL1 protein levels in spinal cord homogenates from *Kif15*^*+/+*^ and *Kif15*^*−/−*^ at 7 dpi. The data was shown as mean ± SD. Statistical analysis was performed using two-tailed unpaired Student’s *t* test, ∗∗*p* < 0.01. n = 4 mice. *B*, schematic diagram of the experiment. Neurons were cultured for 3 days, subjected to injury, and supernatants were collected at 1 dpi for ELISA analysis. *C*, immunofluorescence staining of TUJ1 in E17 cortical neurons isolated from *Kif15*^*+/+*^ and *Kif15*^*−/−*^ mice. Neurons were cultured for 3 days, and then subjected to injury; and further maintained in culture for an additional 2 days prior to immunocytochemical staining. Scale bar = 100 μm. *D*, CX3CL1 levels in culture supernatants from E17 cortical neurons or P1 DRG neurons isolated from *Kif15*^*+/+*^ and *Kif15*^*−/−*^ mice according to the method of *panel B*. The data was shown as mean ± SD. Statistical analysis was performed using two-tailed unpaired Student’s *t* test, ∗*p* < 0.05. *E*, Co-immunoprecipitation (Co-IP) assay demonstrating the interaction between KIF15 and CX3CL1. Neuro-2a cells were transfected with either empty vector (pcDNA3.1–3 × flag, lane 1), full-length CX3CL1 (pcDNA3.1–3 × flag-CX3CL1, lane 2), or truncated CX3CL1 lacking the transmembrane domain (pcDNA3.1–3 × flag-CX3CL1-ΔTM, lane 3). *F*, *left panel*, the fluorescence recovery after photobleaching (FRAP) was performed to assess the fluorescence recovery of mCherry in 293T cells. The *lower panel* shows a magnified view of the *white dashed box* in the *upper panel*, and the *white dashed box* in the *lower panel* indicates the bleached area. *Right panel*, the graph presents the statistical analysis of fluorescence intensity recovery in the bleached region at different time points, n = 15 cells per group. The data were shown as mean ± SD, multiple *t* test performed in different group.

sCX3CL1 release into the culture media was further assessed using an ELISA assay. In the *in vivo* assay, spinal cord tissues from the L4-L6 region of injured wild-type and Kif15 knockout (KO) mice were collected at 7 dpi, gently homogenized in PBS, and the supernatant obtained. The ELISA results revealed a 26.6% decrease in CX3CL1 levels in Kif15 KO mice (12.63–9.27 ng/ml) compared to wild-type mice (Fig. 7*A*). In the *in vitro* assay, Kif15-depleted neurons (from *Kif15*^*−/−*^ mice, E17 cerebral cortex or P1 DRG) were cultured in Xona microfluidic devices. Upon neurite growth into the axonal side, axotomy was induced by electric-pump-controlled vacuum aspiration, and the supernatant from the cell body side was collected 24 h later. The results showed that the concentration of sCX3CL1 released from *Kif15*^*−/−*^ cortical neurons decreased by 28.8% (6.35–4.52 ng/ml) compared to wild-type neurons, and by 34.3% (2.45–1.61 ng/ml) from *Kif15*^*−/−*^ DRG neurons (Fig. 7, *B*–*D*). These data indicate that Kif15 depletion impairs the expression, distribution, and release of soluble CX3CL1 protein. To investigate whether Kif15 interacts with CX3CL1, Co-immunoprecipitation (Co-IP) assays were conducted. The results showed that Kif15 interacts with both full-length CX3CL1 and a truncated version of CX3CL1 (lacking the transmembrane region) in Neuro-2a cells (Fig. 7*E*). Additional Co-IP experiments in E17 spinal neurons are shown in Fig. S4. These results suggest that Kif15, a microtubule plus-end motor, directly affects the membrane localization of CX3CL1. Further, live cell imaging using fluorescence recovery after photobleaching (FRAP) was performed to assess the dynamic transport of CX3CL1. When both Kif15 and CX3CL1 were overexpressed, fluorescence recovery on microtubules after photobleaching was significantly stronger compared to the control group (Figs. 7*F* and S4). This temporal recovery pattern suggests that Kif15 facilitates the dynamic transport of CX3CL1 along microtubules in a time-dependent manner.

### Neurons lacking Kif15 instruct microglia to polarize toward a neuroprotective state

**Figure 8:**
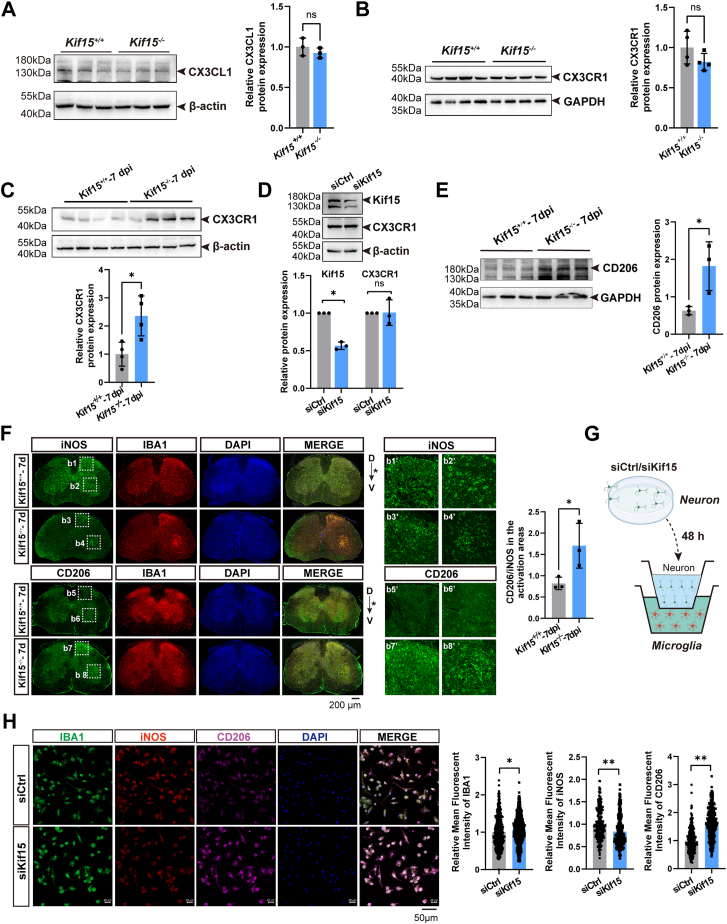
**Neurons lacking Kif15 instruct microglia to polarize towards a neuroprotective state.***A*, *left panel*, Western blot analysis showing the expression levels of CX3CL1 and β-actin in the spinal cord of *Kif15*^*+/*+^ and *Kif15*^*−/−*^ mice. *Right panel*, quantitative analysis of relative CX3CL1 protein expression normalized to β-actin levels. The data was shown as mean ± SD. Statistical significance was determined using a two-tailed unpaired Student's *t* test, with ns indicating no significance. *B*, *left panel*, Western blot analysis of CX3CR1 expression in the spinal cord of *Kif15*^*+/*+^ and *Kif15*^*−/−*^ mice. *Right panel*, quantitative analysis of relative CX3CR1 protein expression normalized to GAPDH levels. The data was shown as mean ± SD. Statistical significance was determined using a two-tailed unpaired Student's *t* test, with ns indicating no significance. *C*, *up panel*, Western blot analysis showing the expression levels of CX3CL1 and β-actin in the spinal cord of *Kif15*^*+/*+^ and *Kif15*^*−/−*^ mice at 7 dpi. *Downpanel*, quantitative analysis of relative CX3CL1 protein expression normalized to β-actin levels. The data was shown as mean ± SD. Statistical significance was determined using a two-tailed unpaired Student's *t* test, with ns indicating no significance. *D*, *up panel*, Western blot analysis showing the expression levels of Kif15, CX3CR1 and β-actin post siKif15 or siCtrl treatment in microglia. *Down panel*, quantitative analysis of relative Kif15 and CX3CR1 protein expression normalized to β-actin levels. The data was shown as mean ± SD. Statistical significance was determined using a two-tailed unpaired Student's *t* test, with ns indicating no significance. *E*, *Left panel*, western blotting assays indicated the expression level of CD206 in spinal cord of *Kif15*^*+/+*^ and *Kif15*^*−/−*^ mice at 7 dpi. *Right panel*, statistical analysis. The data was shown as mean ± SD, two-tailed unpaired Student’s *t* test, (*Kif15*^*+/+*^ group, n = 3 mice, *Kif15*^*−/−*^ group, n = 3 mice), ∗*p* < 0.05. *F*, *Left panel*, immunohistochemistry staining of iNOS and CD206 in spinal cord from *Kif15*^*+/+*^ and *Kif15*^*−/−*^ mice at 7 dpi, scale bar = 200 μm. Panels b1′– b8′ are magnifications of the white squares in panels b1–b8, respectively. *Right panel*, statistical analysis. The *asterisk* represents the nerve injured side, D indicates dorsal, V indicates ventral, the data were shown as mean ± SD. Data were analyzed using two-tailed unpaired Student’s *t* test, ∗*p* < 0.05. *G*, Schematic representation of the experimental design for co-culture of neurons and microglia. Neurons were transfected with siCtrl or siKif15 for 48 h before co-culture with microglia. *H*, *Left panel*, immunofluorescence staining of IBA1, iNOS, CD206, and DAPI in co-cultured neurons and microglia. Scale bar = 50 μm. *Right panel*, statistical analysis of the relative mean fluorescent intensity of IBA1, iNOS, and CD206. The data is presented as mean ± SD. Data were analyzed using two-tailed unpaired Student’s *t* test, ∗*p* < 0.05, ∗∗*p* < 0.01.

Given the critical role of the CX3CL1-CX3CR1 axis in modulating microglial activation (29, 30), the expression of CX3CL1 and CX3CR1 was examined in the spinal cord of adult *Kif15*^*−/−*^ and *Kif15*^*+/+*^ mice under both normal conditions and after SNI. As shown in Figure 8, *A* and *B*, there were no significant changes in the expression of CX3CL1 and CX3CR1 proteins in the spinal cord under normal conditions in either *Kif15*^*−/−*^ or *Kif15*^*+/+*^ mice. However, following sciatic nerve crush in wild-type mice, both CX3CL1 and CX3CR1 proteins increased at 1, 3, and 7 dpi (Fig. S2*A*). Notably, at 7 dpi, CX3CR1 protein levels were elevated by 136% in *Kif15*^*−/−*^ mice compared to wild-type mice (Fig. 8*C*). Given that CX3CL1 was reduced in *Kif15*^*−/−*^ mice (Fig. 6*C*), we examined whether Kif15 depletion increased CX3CR1 expression specifically in microglia. Primary microglia were cultured and treated with siKif15, and Western blot analysis showed no significant changes in CX3CR1 protein expression (Fig. 8*D*). These results suggest that in *Kif15*^*−/−*^ mice, reduced neuronal CX3CL1 may drive increased CX3CR1 expression, enhancing the functional activation of resident microglia.

Next, we assessed CX3CR1 expression through immunostaining, which revealed an increase in CX3CR1 in the anterior horn of the spinal cord gray matter on the injured side. This area coincided with the localization of activated IBA1-positive microglia surrounding the motor neuron cell bodies (Fig. S2, *B* and *C*). To evaluate microglial polarization, M1 and M2 microglia-specific markers (iNOS and CD206, respectively) were examined. Immunostaining results indicated that Kif15 knockout substantially increased CD206 protein levels and the CD206/iNOS ratio (Fig. 8, *E* and *F*), suggesting that Kif15 depletion facilitates microglial polarization toward the M2 phenotype, which is typically associated with anti-inflammatory activity and tissue repair.

Finally, microglia-neuron cocultures were performed to determine whether Kif15 depletion in neurons alters microglial polarization. Neurons treated with siKif15 were subjected to axotomy, placed in the upper chamber, while microglia were seeded in the bottom well. After 24 h of coculture, the microglia were fixed and immunostained for IBA1 (to assess activation), iNOS (for M1 type), and CD206 (for M2 type). The results showed that siKif15-treated neurons induced an increase in activated microglia, which were skewed toward the M2 phenotype (Fig. 8, *G* and *H*).

## Discussion

### Kif15 as a novel negative regulator of peripheral nerve regeneration

Unlike SCI, which typically results in irreversible damage, peripheral nerves are capable of regeneration following SNI, although their cell bodies are localized in the spinal cord. In addition to the supportive role of peripheral Schwann cells in creating a regenerative microenvironment, the responsive changes within the spinal neuronal cell bodies are also critical to this process. With the advent of single-cell sequencing technologies, it has become feasible to investigate gene expression changes across different cell populations in the spinal cord following peripheral nerve injury.

This study identified Kif15, a microtubule-based motor protein, as a novel and essential negative regulator of peripheral nerve regeneration. We demonstrate that genetic deletion of *Kif15* enhances functional recovery after sciatic nerve crush through a dual mechanism: intrinsically promoting neuronal survival and extrinsically driving a pro-regenerative microglial phenotype. The central finding is that Kif15 exerts this regulatory role by controlling the expression and subcellular localization of the neuronal chemokine CX3CL1, thereby modulating neuronal-microglial communication.

The role of KIF15 as a negative regulator of nerve regeneration aligns with an emerging theme in the field of neural regeneration, where many kinesins function not only as cargo transporters but also as critical checkpoints for neuronal growth and repair. For example, KIF11 has been shown to act as a brake on microtubule sliding during axonal extension, with its inhibition promoting faster axon regeneration (31, 32). Similarly, increased expression of KIF21B has been linked to the progression of Alzheimer's disease (33), while its deficiency promotes longer axonal growth in *C. elegans* (34). Together with our findings on KIF15, these studies highlight the importance of a delicate balance in motor protein activity—where both positive and negative regulation of cytoskeletal dynamics and transport are vital for successful nerve regeneration.

Given that initial signals originate from the injured peripheral nerve, which is distant from the neuronal cell body, we hypothesize that motor proteins involved in anterograde and retrograde transport play a pivotal role in this process. Previously, we demonstrated that Kif15 (kinesin-12), a microtubule-associated motor protein, impairs axonal growth and navigation. Depletion of Kif15 significantly enhanced the growth of injured axons, both in cultured neurons and in zebrafish models of SCI (11, 35). In the current study, we used *Kif15*^*−/−*^ mice to model sciatic nerve crush injury and observed improved recovery of the injured nerve in *Kif15*^*−/−*^ mice compared to *Kif15*^*+/+*^ mice. These results suggest that Kif15 deficiency may promote nerve regeneration by altering molecular transport processes.

### snRNA-seq reveals altered cellular communication in Kif15 KO mice

Using snRNA-seq, we confirmed that injured neurons in *Kif15*^*−/−*^ mice exhibited a stronger anti-apoptotic capacity, indicative of enhanced regenerative potential. This finding aligns with our previous studies (35, 36). Additionally, we employed the CellChat method to analyze cell-to-cell communication, focusing on the input and output signals of neurons. The CX3CL1-CX3CR1 signaling axis, a key pathway between neurons and microglia, was found to be weakened in *Kif15*^*−/−*^ mice compared to *Kif15*^*+/+*^ mice. Further investigation into the impact of Kif15 gene deficiency on neuronal CX3CL1 expression revealed that Kif15 plays a role in facilitating the expression and membrane localization of CX3CL1, as well as its release into the surrounding environment.

CX3CL1, also known as Fractalkine, is the sole member of the CX3CL chemokine subfamily and is constitutively expressed on neuronal membranes. Under normal physiological conditions, neuronal CX3CL1 binds to microglial CX3CR1, maintaining microglia in a quiescent state and limiting their activation (37). Following neuronal injury, CX3CL1 undergoes proteolytic cleavage, producing a soluble form (sCX3CL1) that interacts with CX3CR1 on microglia. This interaction triggers signaling pathways that recruit CX3CR1-expressing microglia to the injury site, thereby initiating inflammatory responses. Our study showed that under normal conditions, there was no significant difference in the expression of CX3CL1 and CX3CR1 in the L4-L6 spinal cord between *Kif15*^*+/+*^ and *Kif15*^*−/−*^ mice. However, after SNI, CX3CL1 and CX3CR1 expression notably increased in *Kif15*^*+/+*^ mice, consistent with previous studies indicating that modifications in the CX3CL1-CX3CR1 pathway within the spinal cord can potentiate microglial activation, resulting in sustained neuroinflammatory responses. In contrast, *Kif15*^*−/−*^ mice exhibited lower levels of CX3CL1, in both its membrane-bound and soluble forms.

### The dual role of CX3CL1-CX3CR1 signaling in microglial activation

Current research highlights that microglial activation in models of central nervous system injury, such as sciatic nerve damage, is regulated by the CX3CL1-CX3CR1 signaling pathway. Resident microglia respond rapidly to injury signals, migrating to the damaged neurons and participating in the early stages of injury resolution, including clearing cellular debris and regulating local inflammation. During the initial upregulation of CX3CL1 expression, membrane-bound CX3CL1 interacts with CX3CR1 to help maintain immune homeostasis (38).

Conversely, soluble CX3CL1 recruits microglia from distant sites, guiding them to the injury area. These recruited microglia contribute to persistent inflammation, tissue repair, or scar formation, and in some models, may exhibit an increased capacity to release inflammatory factors such as TNF-α and IL-1β (39). "Recruited" microglia are likely to activate astrocytes and promote glial scar formation, which can inhibit axonal regeneration. In contrast, resident microglia, when CX3CL1 signaling is intact, tend to adopt a neuroprotective phenotype, including functions such as clearing glutamate toxicity and suppressing excessive inflammation (40).

### Kif15 deficiency creates a pro-regenerative environment

Peripheral nerve injury is a common disorder of the nervous system. While such injuries are well known to induce neuronal apoptosis and are associated with neuropathy, the underlying mechanisms remain largely unclear. Most studies have utilized the SNI model to investigate these injuries (41, 42). The ability of nerve cells to regenerate depends on the regeneration of axons after injury; however, the molecular mechanisms driving this process are not fully understood. Spinal microglia can be activated in response to peripheral nerve injury, releasing various mediators that activate adjacent spinal neurons (43). Elimination, suppression, or disruption of microglia activation or microglia-to-neuron signaling, through either genetic or pharmacological means, has been shown to reduce neuropathic pain induced by injury (44).

In this study, functional recovery was notably enhanced in adult *Kif15*^*−/−*^ mice following SNI, as evidenced by a decreased mechanical withdrawal threshold (indicating improved sensory function) and an increased SFI (indicating improved motor function) in *Kif15*^*−/−*^ mice compared to *Kif15*^*+/+*^ mice. Interestingly, spinal microglial activation occurred earlier in *Kif15*^*−/−*^ mice following sciatic nerve crush, but faded more quickly. This finding aligns with our previous study in which SCI in zebrafish with *kif15* morphants in Tg (coro1a:EGFP) zebrafish demonstrated increased recruitment of Coro1a(+) cells to the injury site, followed by a rapid decline in kif15 morphants (45).

The potential influence of Kif15 knockout on microglia-to-neuron signaling was also considered. Although no significant alterations were observed in the distribution of spinal cord neurons, a notable reduction in neuronal apoptosis was detected in Kif15 knockout mice. Our analysis of neuronal apoptosis following nerve injury revealed a substantial decrease in apoptosis in Kif15 knockout mice. Additionally, astrocyte cluster 1, the predominant subpopulation, did not show changes in A1/A2 astrocyte-associated marker gene expression, which may explain the absence of effects on astrocyte activation after Kif15 depletion (Fig. S3). Interestingly, microglial activation in Kif15 knockout mice occurred earlier than in wild-type mice following SNI, and the proportion of microglia was significantly increased in Kif15 knockout mice compared to wild-type controls. Further analysis and validation revealed that Kif15 knockout promotes microglial M2 polarization. This transient microglial activation in Kif15-deficient mice may be attributed to the disruption of axonal transport and alterations in the CX3CL1-CX3CR1 neuro-immune axis. On one hand, loss of Kif15 could enhance retrograde transport of injury-sensing cargos to the neuronal soma, rapidly triggering microglial recruitment at the injury site, as some studies suggest that dynein-mediated cargo transport is enhanced following kinesin-1 autoinhibition (46). On the other hand, reduced CX3CL1-CX3CR1 signaling may dampen sustained pro-inflammatory microglial activation, leading to a swift resolution of microglial reactivity and promoting a shift towards an anti-inflammatory state favorable for tissue repair (29).

### Unresolved questions and future directions

A critical question arising from our data is how Kif15, a cytoskeletal motor, regulates gene expression. Recent studies investigating kinesin-mediated gene expression offer a framework for understanding KIF15's potential role. KIF17 has been shown to directly modulate transcription by shuttling transcription factors between the nucleus and cytoplasm (47, 48). Swarnkar *et al.* demonstrated that KIF5C functions as a molecular motor transporting ribonucleoprotein complexes (RNPs) to distal neuronal compartments, thereby facilitating local translation (49). While our data clearly show that *Kif15* knockout reduces *Cx3cl1* mRNA levels, the precise mechanism remains unclear. We hypothesize that Kif15 may be involved in the anterograde transport of certain RNPs, which are complexed with *Cx3cl1* mRNA and essential for the translational regulation of CX3CL1. Alternatively, Kif15-mediated microtubule dynamics could indirectly influence nuclear signaling and gene expression programs in response to injury. This presents an exciting avenue for future research, potentially revealing a broader role for Kif15 in regulating the neuronal transcriptome during stress and repair.

Beyond transcriptional regulation, our biochemical and cell biological evidence strongly supports a direct role for Kif15 in the post-translational processing of the CX3CL1 protein. The Co-IP interaction between Kif15 and both full-length and truncated CX3CL1 suggests that Kif15 functions as either a transport motor or a scaffolding protein, facilitating the trafficking of CX3CL1 to the plasma membrane. The FRAP assay clearly demonstrates that Kif15 assists in the transport of CX3CL1 along microtubules to the cell membrane. The significant reduction in membrane-localized and soluble CX3CL1 upon Kif15 depletion further highlights its critical role in maintaining the proper spatial distribution and functional integrity of this key signaling molecule. This positions Kif15 at a key intersection, directly linking intracellular transport machinery to the output of a vital neuro-immune signal.

The functional consequence of attenuating the CX3CL1-CX3CR1 axis in Kif15 knockout mice is a shift in microglial response. Early, transient activation and pronounced M2 polarization, particularly in the expanded microglial cluster 3 with its synaptic remodeling signature, created a microenvironment conducive to repair. An important consideration is the relative contribution of intrinsic neuronal resilience *versus* the altered microglial phenotype to overall functional recovery. While our coculture experiment confirms that Kif15-deficient neurons can instructively drive microglia toward a protective state, enhanced anti-apoptotic signaling in neurons likely works in tandem with this extrinsic factor.

As a microtubule-based kinesin, Kif15 facilitates the transportation and localization of CX3CL1 in neurons and regulates microglial activation by binding to its receptor, CX3CR1, thereby maintaining communication between neurons and microglia. We propose that neuronal Kif15 regulates the amount of membrane-bound CX3CL1, which in turn governs microglial responses of "eat me" or "save me" following nerve injury. Future studies employing microglia-specific Cx3cr1 knockout or inhibition in the Kif15 knockout background could help elucidate the precise contribution of this communication axis to the regenerative phenotype.

### Conclusion and working model: Kif15 as a master coordinator of neuro-immune communication

**Figure 9:**
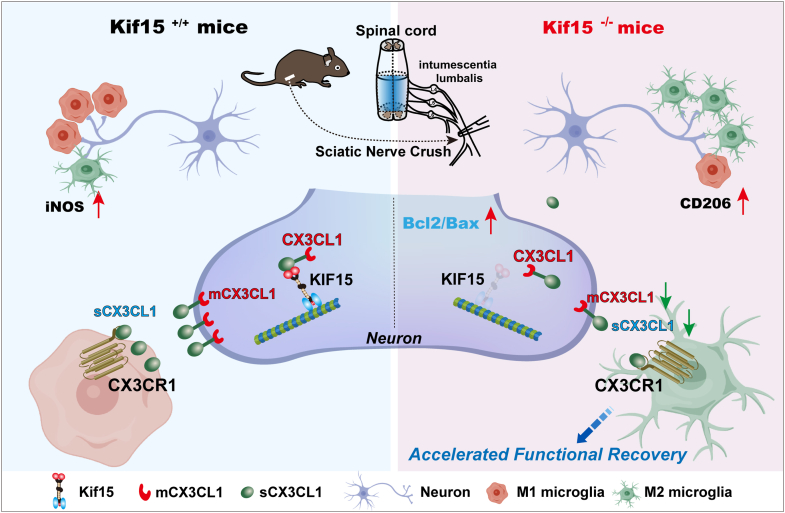
**A model for Kif15-mediated neuronal-microglial communication during nerve repair.** This study uncovers a novel role for the motor protein Kif15 as a regulator of nerve regeneration. In wild-type mice (*left*), Kif15 facilitates CX3CL1-dependent signaling, maintaining microglial activation and limiting repair processes. In contrast, Kif15 knockout mice (*right*) exhibit disrupted CX3CL1 expression and localization, impairing neuron-microglia communication. This disruption leads to a more protective microenvironment, marked by reduced neuronal apoptosis and a shift of microglia toward an M2, pro-regenerative phenotype, ultimately resulting in accelerated functional recovery.

In conclusion, we have expanded the functional role of Kif15 from a regulator of microtubule dynamics and axonal growth to a key coordinator of neuro-immune communication (Fig. 9, created with bioGDP.com). By influencing both the production and delivery of CX3CL1, Kif15 emerges as a key determinant of the neuronal response to injury and the subsequent activation of the innate immune system. Targeting the Kif15-CX3CL1 pathway may offer a novel therapeutic strategy for promoting nerve repair by enhancing neuronal health and harnessing the protective power of microglia.

## Experimental procedures

### Animals

Kinesin-12 (*Kif15*) knockout mice were constructed using the CRISPR/Cas9 system and provided by GemPharmatech. The mouse *Kif15* gene (GenBank: NM_010620.1) is located on chromosome 9 and contains 35 exons with the start codon in the first exon and the stop codon in the last exon. To construct the *Kif15* knockout mice, mice with a conditional *Kif15* allele were first created (B6/JNju*-Kif15*^*em1Cflox*^*/Nju*), with Exon 2 and Exon 3 flanked with loxP. B6/JNju*-Kif15*^*em1Cflox*^*/Nju* were then mated with Tg (CMV-cre)1Cgn to form a *Kif15* null allele (*Kif15*^*−/−*^). The gene knockout founders were genotyped by PCR followed by DNA sequencing analysis. The homozygous mutant mice were generated by intercross of heterozygous mutants. All WT and knockout mice had a C57BL/6J background. The schematic representation of the construct is given in Figure 1*A*.

The mice were housed in a temperature-controlled room at 25 ± 2 °C at a specific pathogen-free facility with a light/dark cycle of 12 h light and 12 h dark. All studies reported in this article have been submitted to the Animal Experiment Ethics Committee of Nantong University and have been approved by the Animal Protection and Use Committee of Nantong University (P20250228-038).

### Genotyping

Mutant mice genotyping was performed by PCR amplification of genomic DNA extracted from 3-week-old mouse tail tips. The PCR primers for the *Kif15* mutant allele were: (Forward) 5′- aggattctggaggcagacaggt-3′ and (Reverse) 5′-gggatggagttcatttagcagaca -3′, and the PCR product was 634 bp. PCR primers for the wild-type *Kif15* allele were: (Forward) 5′- aatataatgggctgagatagagt-3′ and (Reverse) 5′- tatggaccctacacaaattgc-3′, and the PCR product was 219 bp.

### Injury model of the sciatic nerve crush

The surgical procedure of sciatic nerve crush was performed according to a modification of the pervious reported method (50). Briefly, adult mice(10-week-old) were anesthetized by an intraperitoneal injection of pentobarbital sodium and weighed, and the sciatic nerve was identified and lifted through an incision on the lateral aspect of the mid-thigh of the right hind leg. The right lateral sciatic nerve was crushed with a 2-mm hemostat three times (10 s each time) with an interval of 10 s. For consistency of the procedure, all operations of sciatic nerve crush were done by the same well-experienced person. The surgical incisions were closed, and the animals were returned to their cages.

### Behavioral analysis

Sciatic behavioral function is assessed using the sciatic functional index (SFI) which is a generally used test to assess behavioral recovery and return of function after sciatic nerve injury (51). Briefly, the hind paws of each animal are moistened with chemically synthesized red paint and the animals are allowed to walk unassisted along a 6 cm wide and 70 cm long corridor lined with white paper. The prints chosen for measurements are complete.

To determine sensitivity to mechanical force, the von Frey test was used (52). Mice were placed in a rectangular chamber on an elevated mesh grid, and the plantar surface was stimulated with a von Frey filament, which would bend upon reaching the surface of the hindpaw. We stimulated their hindpaws with a series of von Frey hairs with logarithmically increasing stiffness (0.02–2.56 g). Each paw of the mouse was stimulated for 10 trials, with an intertrial interval of at least 10 minutes, and the latency to withdrawal was averaged over all 20 trials.

To measure the error rate of mice after sciatic nerve injury, we used ‘over-ground walking’ as the primary task, in which mice had to walk in one direction in a gangway with a length of 100 cm, a width of 5 cm and a wall height of 5 cm. Their movement path was recorded with a camera and percentage performance error was calculated for each mouse during a length of 80 cm.

To measure motor activity, the rotarod test was used. Mice were first trained on the rotarod for a 120 s training session at a constant speed of 4 rpm. Mice were then tested a day later on the rotarod while accelerating the rod at an increase of 5 rpm/min. Each mouse performed 3 trials with an intertrial interval of at least 10 min, and latency to fall was averaged over all three trials.

### Single-nuclei sequencing

After 7 days of sciatic nerve injury, the 5 mm long spinal cord lumbar segments on the injured side were collected and sent to Singleron Biotechnologies Company for single nucleus sequencing. Briefly, prior to dissociation, tissue samples were washed with pre-cooled PBSE (PBS buffer containing 2 mM EGTA) for three times. Add 50ul pre-cooled Nuclear Extraction Buffer (Singleron) and use surgical scissors cut the tissue to mince. Then, add 950 ul Nuclear Extraction Buffer again, mix well, and let stand on ice for 5 min. Filter the sample into 50 ml centrifuge tube and flush the filter screen with 9 ml PBSE. After centrifugation with 200rcf for 2 min, take 9 ml supernatant and put it into a new 15 ml centrifuge tube. Then centrifuged at 500rcf for 5 min, and resuspended with 50 μ l precooled PBS mix after abandoning the supernatant. Add DAPI staining solution (Sigma) and react for 2 min on ice in the dark. Finally, the pre-cooled PBS was used to adjust the nucleus concentration to 3 to 4 × 105 nuclear per mL for use. Single-cell nuclei were then loaded onto a microfluidic chip (GEXSCOPE Single Cell RNA-seq Kit, Singleron Biotechnologies) and scRNA-seq libraries were constructed according to the manufacturer’s instructions (Singleron Biotechnologies). The resulting scRNA-seq libraries were sequenced on an Illumina HiSeq X10 instrument with 150 bp paired end reads.

### Primary analysis of raw read data

Raw reads were processed with fastQC and fastp to remove low quality reads. Poly-A tails and adaptor sequences were removed by cutadapt. After quality control, readings were mapped to the reference genome GRCm38 (mm10) using STAR. Gene counts and UMI counts were acquired by featureCounts software. Expression matrix files for subsequent analyses were generated based on gene counts and UMI counts.

### Quality control and cell-type clustering

Cells were filtered by gene counts below 200 and the top 2% gene counts and the top 2% UMI counts. Cells with over 20% mitochondrial content were removed. A total of 26,603 filtered cells were used for further bioinformatic analysis. We used Seurat v 3.1.2 to first normalize expression matrices by function NormalizeData and ScaleData. Then FindVariable function was applied to select the top 2000 variable genes and perform principal component analysis. The first 20 principal components and resolution 1.0 were used with FindClusters function to generate 24 cell clusters. To assign nine cell types to each cluster, we scored each cluster by the normalized expressions of the following canonical markers: Neurons (*Syt1, Snap25, Grin1, Sst*), Astrocytes (*Aqp4, Ntsr2, Aldoc, Slc1a2*), Microglial cells (*Hexb, P2ry12, C1qb, Siglech, Cx3cr1*), Oligodendrocyte progenitor cells (*Pdgfra, Bcan, Sox6, Vcan, Tnr, Ptprz1*), Oligodendrocytes (*Mobp, Mog, Mag, Plp1*), Endothelial cells (*Ly6c1, Cldn5, Vwf, Eng*), Ependymal cells (*Nnat, Dnah12, Spag16*), Schwann cells (*Mpz, Pmp22, Prx, Cryab*), Meningeal cells (*Vtn, Igfbp2, Dcn, Ptgds*).

### Differentially expressed genes (DEGs) and Cell type annotation

Genes expressed in more than 10% of the cells in a cluster and with average log (Fold Change) of greater than 0.25 were selected as DEGs by Seurat v3.1.2 FindMarkers based on Wilcox likelihood-ratio test with default parameters. The cell type identity of each cluster was determined with the expression of canonical markers found in the DEGs using SynEcoSys database. Heatmaps/dot plots/violin plots displaying the expression of markers used to identify each cell type were generated by Seurat v3.1.2 DoHeatmap/DotPlot/Vlnplot.

### GO/KEGG enrichment and cellchat analysis

To investigate the potential functions of Kif15, the Gene Ontology (GO) and Kyoto Encyclopedia of Genes and Genomes (KEGG) analysis were used with the “clusterProfiler” R package v3.16.1. Pathways with p_adj value less than 0.05 were considered as significantly enriched. Gene Ontology gene sets including molecular function (MF), biological process (BP), and cellular component (CC) categories were used as reference. The chord diagram showing interactions sending from neurons to all other cells was generated using netVisual_chord_gene in cellchat (version 1.5.0, https://github.com/sqjin/CellChat).

### Cellchat analysis

The single cell nuclei sequencing data were packaged into a Seurat object using Seurat 5.1.0 (53), and analyzed using CellChat 1.5.0 (22). The chord diagrams of signaling pathways were generated using netVisual_chord_gene from CellChat following the tutorial at https://github.com/sqjin/CellChat/blob/master/tutorial/Comparison_analysis_of_multiple_data-sets.html.

### Cell culture and transfection

Primary microglia of postnatal day one rat brain were prepared as previously described (54), cultured in Dulbecco's Modified Eagle Medium/Nutrient Mixture F-12 (DMEM/F12, corning, # 10-092-CV), supplemented with 10% fetal bovine serum (FBS; Biological Industries, # 04-001-1ACS), 0.5 mM glutamine (Beyotime, #C0212), and 1% penicillin-streptomycin (Beyotime, #C0224), and incubated in a humidified atmosphere of 95% air and 5% CO_2_ at 37 °C.

The Mouse neuro-2a (N2a) and BV2 microglial cell lines were procured from the American Type Culture Collection (ATCC) and were cultivated following standard protocols. Microglia/N2a/BV2 cells were used for siRNA or plasmids transfection by a NEPA21 electrical transfection instrument (55). The siRNAs were synthesized by GENCEFE Biotech, Wuxi, China. Their sequences are listed in Table 1.

**Table 1:** The sequences of primers and siRNAs

Name	Sense (5′-3′)	Antisense (5′-3′)
*Gapdh*	tgccactcagaagactgtgg	caacggatacattgggggta
*Kif15*	tcgtccagacagccaaca	ctcgctcattctgtggca
*Cx3cl1*	gcagatccccagaaactgag	ggaggccttctaccatttcc
*Cx3cr1*	tttgccggggaaaagttcag	ccatctccctcgcttgtgta
*18s rRNA*	cgcggttctattttgttggt	cctccgactttcgttcttga
*IL-1β*	tgtcttggccgaggactaag	tgggctggactgtttctaatg
*TGF-β*	cctatttaagaacacccacttt	tcctgaataatttgaggttgag
Control (Ctrl) siRNA	uucuccgaacgugucacgutt	acgugacacguucggagaatt
Mouse Kif15 siRNA	cagcuauaauugcaaaugutt	acauuugcaauuauagcugtt

### Western blotting

Cells or tissues were lysed to extract total proteins, and protein concentrations in the total cell extracts were measured using the bicinchoninic acid protein (BCA) assay. Total protein samples (20 μg) were separated by sodium dodecyl sulphate–polyacrylamide gel electrophoresis, and membranes were incubated overnight at 4 °C with the following primary antibodies: anti- Kif15 antibody (Proteintech, Cat: #55407-1-AP, 1:2000), anti-CD206 (Proteintech, Cat: #60143-1-Ig,1:1000), anti-CX3CR1 (Immunoway, Cat: # YT5112, 1:1000), CX3CL1 (affinity, Cat: #DF12376, 1:1000), anti-Bcl2 (Cell Signaling Technology, Cat: # 3498T, 1:1000), anti-Bax (Cell Signaling Technology, Cat: # 2772T, 1:1000), anti-Flag (Proteintech, Cat: # 20543-1-AP,1:20000), anti-GAPDH (Proteintech, Cat: #60004-1-Ig, 1:5000) and anti-β-actin (Proteintech, Cat: # 60008-1-Ig, 1:5000). After washing with Tris-buffered saline and 0.1% Tween 20, membranes were incubated with horseradish peroxidase-conjugated secondary anti-rabbit or anti-mouse antibodies at room temperature for 2 h. For visualization, the immunoreactive bands were treated with a chemiluminescence solution (Tanon, Cat: #180-5001) and detected using X-ray films. The optical density values of the target protein bands were quantified using Multi Gauge software (FujiFilm Corp. Life Science Division) and normalized to the GAPDH/β-actin loading Ctrl.

### Immunohistochemistry

Mice were perfused with 4% paraformaldehyde (Macklin,#P80453) and the 0.5 cm spinal cord segments of lumbar intumescence and sciatic nerve were collected and cryoprotected in 30% sucrose. Tissue sections (20 μm) were then prepared by cryostat sectioning and were treated with blocking buffer (0.4% BSA, 5% goat-serum, and 0.2% Triton-X 100 in PBS) for 120 min at 37 °C. Primary antibodies for GFAP (Abcam, Cat: # ab4674, 1:200), IBA1(Abcam, Cat: # ab178846, 1:200), CX3CL1 (affinity, Cat: #DF12376, 1:100), CX3CR1 (Immunoway, Cat: # YT5112, 1:200), CD206 (Proteintech, Cat: #60143-1-Ig,1:200), iNOS (abcam, Cat: # ab178945,1:400), Tuj1 (Biolegend, Cat: # 801202,1:1000), SCG10 (Proteintech, Cat: # 10586-1-AP, 1:500) and NeuN (Abcam, Cat: #ab104224, 1:200) were incubated overnight at 4 °C in blocking buffer. After rinse in PBS, the corresponding secondary antibodies were incubated for 120 min at room temperature. All tissues were counterstained with DAPI (Sigma, Cat: #D9542,1:4000). The tissues were then washed and mounted. Fluorescence images were obtained with a Zeiss fluorescence microscope.

### Sciatic nerve tissue clearing

Following mouse perfusion, the sciatic nerve was extracted, stripped of epineurium, and fixed in 4% PFA at 4 °C overnight. After washing in 0.01 M PBS, the nerve was permeabilized and blocked in a permeabilization/blocking solution for 16 h at 4 °C. It was then incubated with a primary antibody (rabbit anti-TUJ1, 1:800) for 5 days at 4 °C. Following extensive PBS washes to remove unbound primary antibody, the nerve was incubated with a secondary antibody (Cy3-conjugated goat anti-rabbit IgG, 1:400) and Hoechst 33342 (1:4000) for 48 h at 4 °C. After further PBS washes, the nerve was cleared with Visikol HISTO-1 and HISTO-2 and imaged using confocal microscopy.

### RNA extraction and real-time quantitative PCR

Total RNA was extracted using TRIzol reagent (Cat: # 15596-026, Thermo Fisher Scientific), and cDNA was synthesized using HiScript III first Strand cDNA Synthesis Kit (+gDNA wiper) (Vazyme, Cat: #R312-02). Real-time quantitative PCR (qPCR) was performed with TB Green Premix Ex Taq (Takara, Cat: # RR820A) using the qTower3G Fluorescent quantitative PCR instrument (Jena, Germany). The primers were synthesized by the General Biol (Anhui) Co., Ltd (Anhui, China). Gapdh was used as the internal control. Primers are listed in Table 1.

### Microfluidic assays

Primary cortical neurons were isolated from embryonic day 17 (E17) mouse embryos, and dorsal root ganglion (DRG) neurons from postnatal day 1 (P1) pups. Cells from both tissues were genotyped as either *Kif15*^*+/+*^ or *Kif15*^*−/−*^. Neurons were plated at equal densities on microfluidic devices (Xona Microfluidics, SND150; 150 μm microgrooves) that permit fluidic isolation of somatic and axonal compartments. After 3 days *in vitro*, complete medium was replaced with neurobasal medium lacking growth factors, and axotomy was performed by aspirating the axonal compartment to induce injury. Conditioned medium was collected from the somatic compartment 24 h post-axotomy, cleared by centrifugation (1000 *g*, 10 min, 4 °C), and stored at −80 °C until CX3CL1 levels were quantified by ELISA kit according to the manufacturer’s protocol.

### Enzyme-linked immunosorbent assay

Cell-free supernatants derived from cortical and DRG neurons of *Kif15*^*+/+*^ and *Kif15*^*−/−*^ mice were collected before and after axotomy; homogenates of L4–L6 spinal cord segments were prepared 7 days after SNI from the same genotypes. All samples were cleared by centrifugation (1000 *g*, 20 min, 4 °C), and CX3CL1 levels in the resultant supernatants were quantified with a commercial mouse ELISA kit (Elabscience, Cat: # E-EL-M0267) according to the manufacturer’s instructions.

### Co-immunoprecipitation

Full-length CX3CL1 (CX3CL1-FL) and a transmembrane-deletion mutant (CX3CL1-ΔTM) were PCR-amplified and inserted into pcDNA3.1 to 3 × Flag. Empty pcDNA3.1 to 3 × Flag served as vector control. Neuro-2a cells were electroporated with 3 μg of each plasmid. Forty-eight hours post-transfection, cells were lysed in IP buffer (Pierce). Protein concentrations were normalized to 1 mg/ml (BCA assay). Lysates (500 μg) were incubated overnight at 4 °C with 50 μl of Pierce Anti-DYKDDDDK Magnetic Agarose (Thermo, Cat: # A36797) on a 10-rpm rotator. Beads were washed, eluted in 50 μl IP buffer plus 10 μl 6 × loading buffer, and analyzed by Western blot with anti-Flag (CX3CL1) and anti-Kif15 antibodies.

### Live cell imaging for FRAP

Referring to our previous study (14), the 293T cell line was co-transfected with pmChery-N1-CX3CL1 and peGFP-N1-Kif15 plasmids for 47h, followed by Hoechst staining for 20 min in an incubator at 37 °C (1:1000, C1011, Beyotime) and washing three times with PBS. The cells were stained at 37 °C for 40 min according to the instructions of the Tubulin-Tracker Deep Red Staining Kit for Living Cells (C2215S, Beyotime), and the staining solution was discarded. Subsequently, the cells were washed three times with preheated staining solution containing 1% staining enhancer, High-glucose DMEM complete medium (containing 10% FBS, 1% penicillin-streptomycin, and amphotericin) was added, and live cells were captured by confocal microscopy. GFP and mCherry fluorescence signals were bleached with 100% 488 nm and 561 nm laser, respectively, and 20 images were taken with the same parameters after bleaching. The fluorescence recovery intensity (R) at each time point (F30,F60 and F90) was equal to the fluorescence intensity of the bleated area at that time point((t = 30s, 60s and 90s)) minus the same field immediately after bleaching (F0) divided by the difference between the initial fluorescence intensity of the field before photobleaching (Fi) and F0 That is, Rt= (Ft-F0)/(Fi-F0) (56).

### Statistical analysis

All The data was shown as mean ± SD from at least three independent experiments. Analyses were performed with GraphPad Prism 10.1 (GraphPad Software, San Diego, CA, USA). Statistical significance was evaluated by one-way ANOVA followed by Tukey’s *post hoc* test, or by two-way ANOVA when data were influenced by two factors. Unpaired two-tailed Student’s *t* tests were used for two-group comparisons. Differences were considered significant at *p < 0.05.*

## Data availability

The datasets generated during and/or analyzed during the current study are available from the corresponding author on reasonable request.

## Supporting information

This article contains supporting information.

## Conflict of interest

The authors declare that they have no conflicts of interest with the contents of this article.
