# Supplementary material for: Kif15 orchestrates neuronal–microglial communication via CX3CL1 to impede nerve regeneration
Source: J Biol Chem. 2026 Apr 28;302(6):113090. doi: 10.1016/j.jbc.2026.113090 (PMC13224079; doi:10.1016/j.jbc.2026.113090)
Supplement: Supplementary Material [file mmc1.docx]

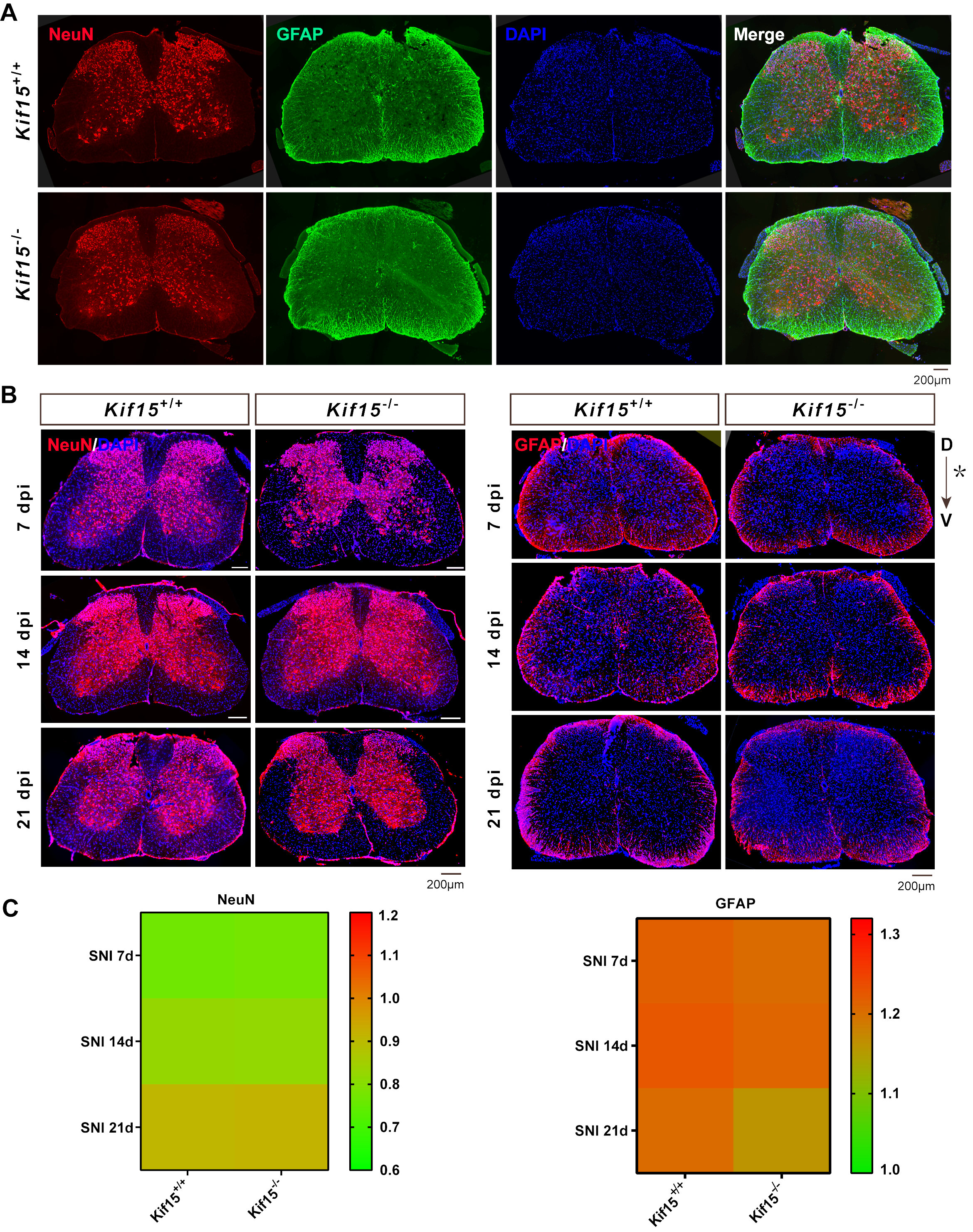
 **Supplemental Figure 1. Comparative analysis of neuronal and glial marker expression in *Kif15^+/+^* and *Kif15^-/-^* mice following sciatic nerve injury (SNI).**

**A**, Representative immunofluorescence images showing the expression of neuronal marker NeuN (red), glial fibrillary acidic protein (GFAP, green), and nuclear marker DAPI (blue) in the lumbar spinal cord segments of *Kif15^+/+^* and *Kif15^-/-^* mice. Scale bar = 200 µm.

**B**, Immunohistochemistry staining of NEUN or GFAP (red) and DAPI (blue) in spinal cord lumbar segments at 7, 14, 21 dpi. The asterisk indicates the nerve-injured side. Dorsal (D) and ventral (V) orientations are marked. Scale bar = 200 µm.

**C**, Heat map of NeuN and GFAP expression in spinal cord lumbar segments at 7, 14, 21 dpi. The ratio of average fluorescence intensity at the nerve injured side and the contralateral side, were used to plot the heatmap. Red color represents high gene expression and green low expression.


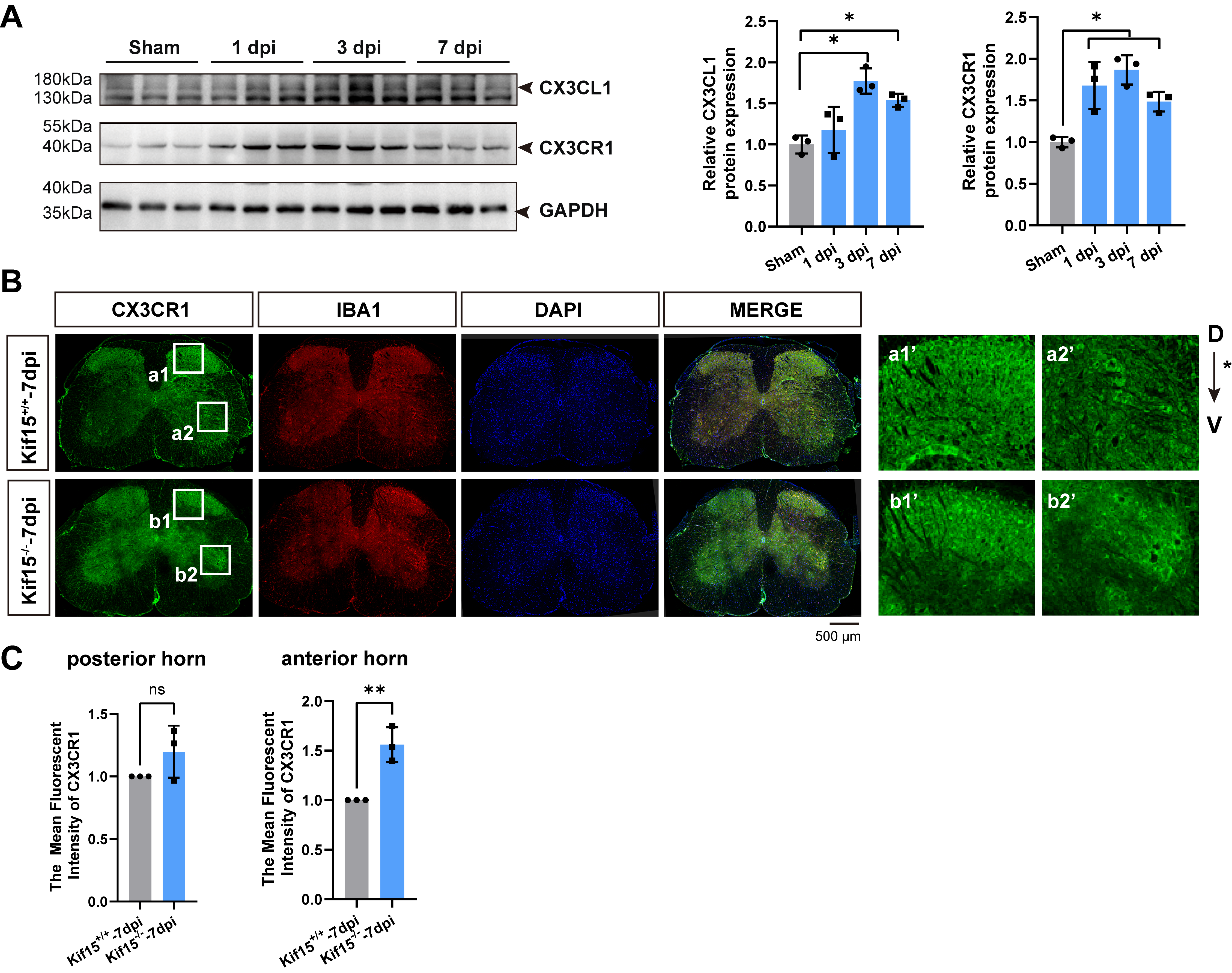
 **Supplemental Figure 2. Expression of CX3CR1 in the spinal cord of Kif15^+/+^ and Kif15^-/-^ mice after sciatic nerve injury**

**A,** Left panel, time course analysis of CX3CL1 and CX3CR1 expression in the spinal cord of Kif15^+/+^ mice after sham or nerve injury (1, 3, and 7 days post-injury, dpi). Right panel, quantitative analysis of relative CX3CL1 and CX3CR1 protein expression at different time points post-injury. Data were analyzed using one-way ANOVA followed by Tukey’s post hoc test, * p < 0.05.

**B**, Representative immunofluorescence images showed the expression of CX3CR1 (green), IBA1 (red), and DAPI (blue) in the posterior and anterior horns of the spinal cord. Panels (a1’, a2’, b1’, b2’) are magnified views of the panels (a1, a2, b1, b2). Scale bar = 500 µm.

**C**, Quantification of the mean fluorescent intensity of CX3CR1 in the posterior and anterior horns of the spinal cord. Data are presented as mean ± SEM. Statistical significance was determined using an unpaired t-test: ns (not significant), **p < 0.001.


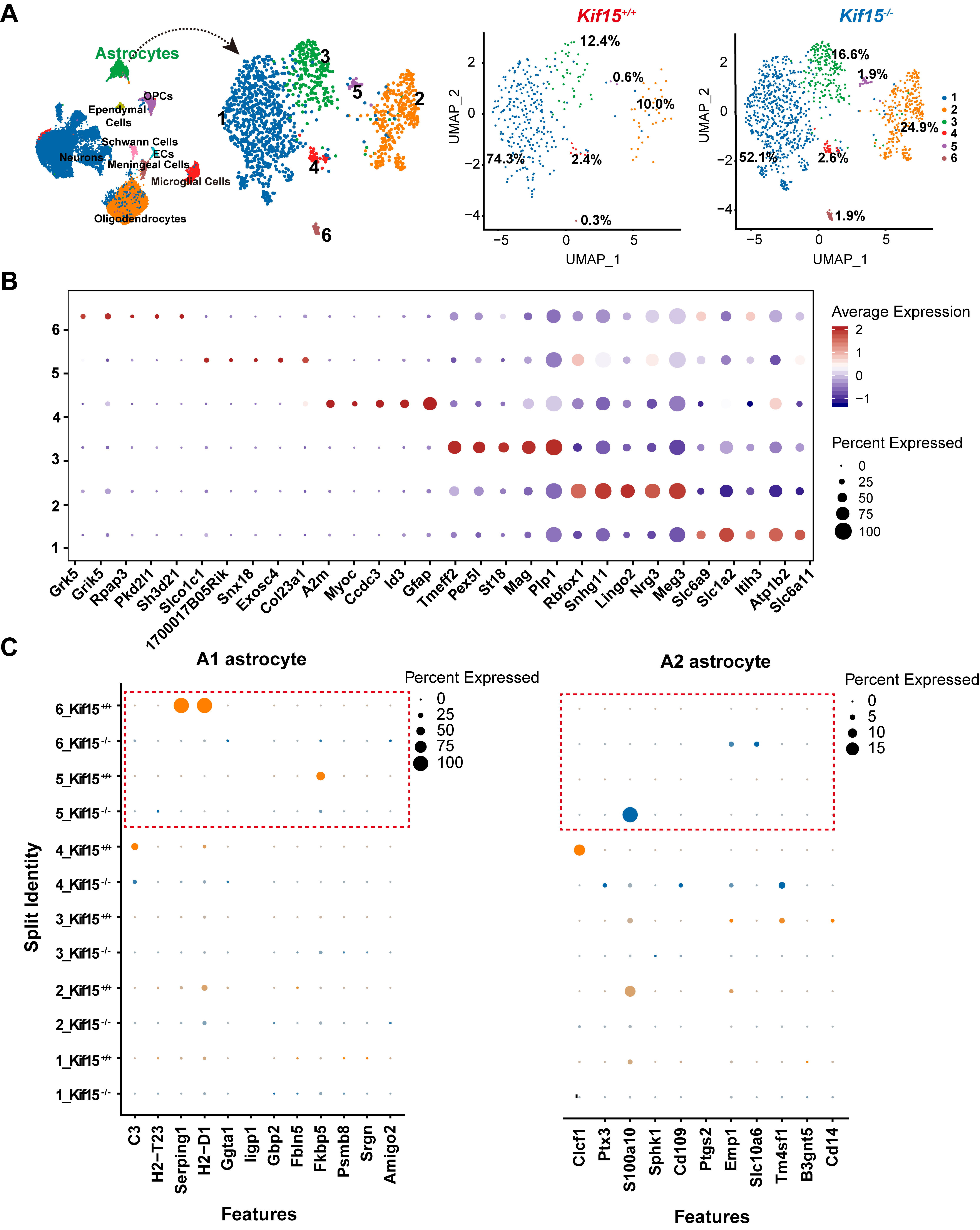
 **Supplemental Figure 3. Diverse astrocytic subpopulations converged between Kif15^+/+^ and Kif15^-/-^ mice post SNI.**

**A**, Left panel, UMAP plot showing six astrocyte clusters (subtypes). Middle panel shows the proportion of each astrocyte subtype in *Kif15^+/+^* mice after SNI 7d. Right panel shows the proportion of each astrocyte subtype in *Kif15^-/-^* mice after SNI 7d.

**B**, Dotplot illustrating the normalized mean expression of top 5 signature genes for each astrocyte cluster.

**C**, Dotplot illustrating the normalized mean expression of A1/A2 astrocyte marker genes among six subtypes between *Kif15*^+/+^ mice and *Kif15*^-/-^ mice.


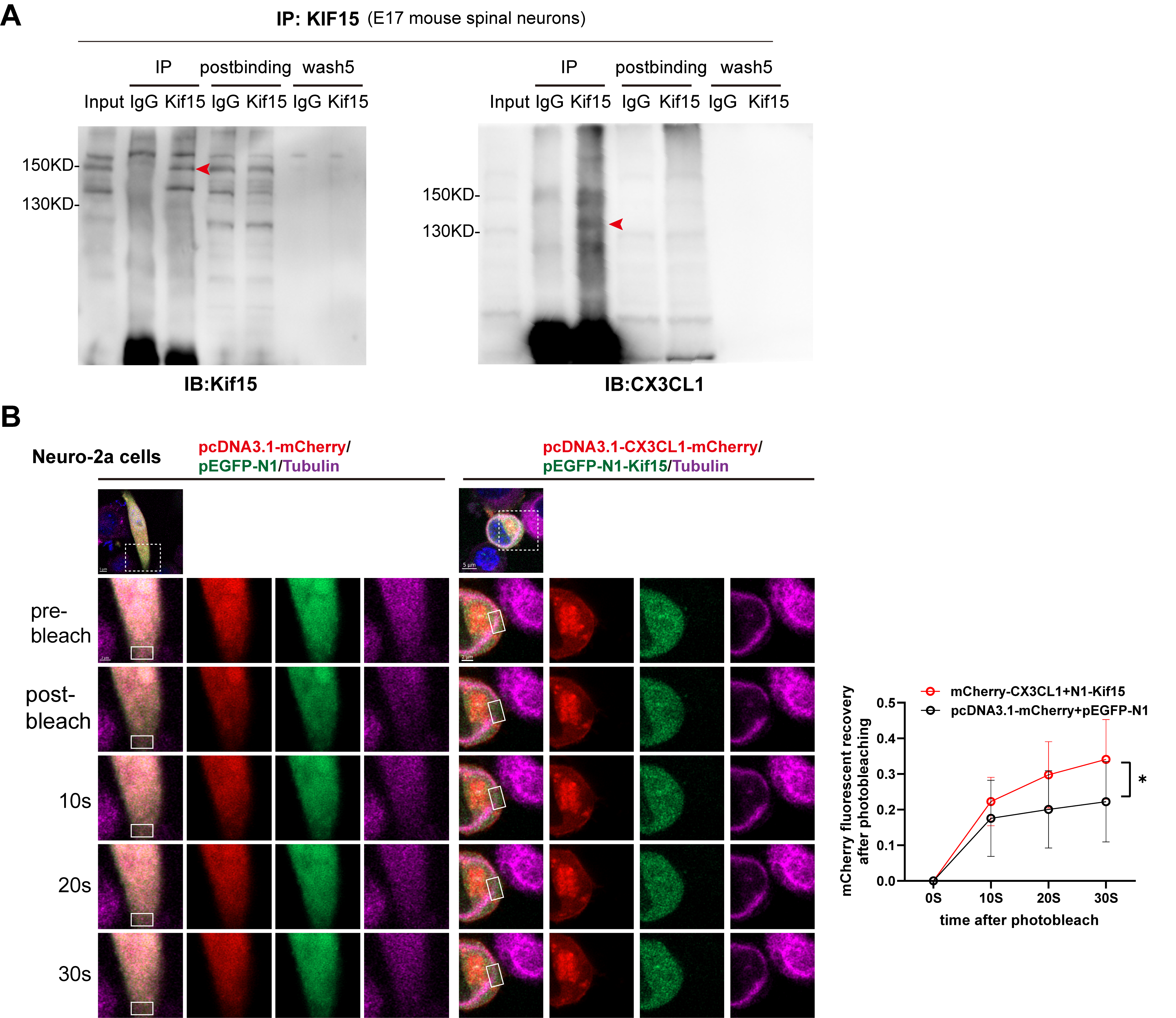


**Supplemental Figure 4. KIF15 mediates the transport of CX3CL1 on microtubules**

**A**, Co-immunoprecipitation (Co-IP) assay demonstrating the interaction between KIF15 and CX3CL1 in E17 spinal neurons.

**B**, Left panel, the fluorescence recovery after photobleaching (FRAP) was performed to assess the fluorescence recovery of mCherry in Neuro-2a cells. The lower panel shows a magnified view of the white dashed box in the upper panel, and the white dashed box in the lower panel indicates the bleached area. Right panel, the graph presents the statistical analysis of fluorescence intensity recovery in the bleached region at different time points, n = 6 cells per group. The data was shown as mean ± SD, multiple t test performed in different group, * *p*<0.05.
